# Transcriptomic dissection of tongue squamous cell carcinoma

**DOI:** 10.1186/1471-2164-9-69

**Published:** 2008-02-06

**Authors:** Hui Ye, Tianwei Yu, Stephane Temam, Barry L Ziober, Jianguang Wang, Joel L Schwartz, Li Mao, David T Wong, Xiaofeng Zhou

**Affiliations:** 1 Center for Molecular Biology of Oral Diseases, College of Dentistry, University of Illinois at Chicago, Chicago, IL, USA; 2 Department of Biostatistics, Rollins School of Public Health, Emory University, Atlanta, GA, USA; 3 Department of Thoracic/Head and Neck Medical Oncology, University of Texas, MD Anderson Cancer Center, Houston, TX, USA; 4 Department of Head and Neck Surgery, Institut Gustave-Roussy, Villejuif, France; 5 Department of Otorhinolaryngology – Head and Neck Surgery, University of Pennsylvania Health System, Philadelphia, PA, USA; 6 Department of Oral & Maxillofacial Surgery, the Second Affiliated Hospital of Sun Yat-sen University, Guangzhou, China; 7 Oral Medicine & Diagnostic Sciences, College of Dentistry, University of Illinois at Chicago, Chicago, IL, USA; 8 Graduate College, UIC Cancer Center, University of Illinois at Chicago, Chicago, IL, USA; 9 Dental Research Institute, School of Dentistry, Jonsson Comprehensive Cancer Center, University of California at Los Angeles, Los Angeles, CA, USA; 10 Guanghua School & Research Institute of Stomatology, Sun Yat-sen University, Guangzhou, China

## Abstract

**Background:**

The head and neck/oral squamous cell carcinoma (HNOSCC) is a diverse group of cancers, which develop from many different anatomic sites and are associated with different risk factors and genetic characteristics. The oral tongue squamous cell carcinoma (OTSCC) is one of the most common types of HNOSCC. It is significantly more aggressive than other forms of HNOSCC, in terms of local invasion and spread. In this study, we aim to identify specific transcriptomic signatures that associated with OTSCC.

**Results:**

Genome-wide transcriptomic profiles were obtained for 53 primary OTSCCs and 22 matching normal tissues. Genes that exhibit statistically significant differences in expression between OTSCCs and normal were identified. These include up-regulated genes (MMP1, MMP10, MMP3, MMP12, PTHLH, INHBA, LAMC2, IL8, KRT17, COL1A2, IFI6, ISG15, PLAU, GREM1, MMP9, IFI44, CXCL1), and down-regulated genes (KRT4, MAL, CRNN, SCEL, CRISP3, SPINK5, CLCA4, ADH1B, P11, TGM3, RHCG, PPP1R3C, CEACAM7, HPGD, CFD, ABCA8, CLU, CYP3A5). The expressional difference of IL8 and MMP9 were further validated by real-time quantitative RT-PCR and immunohistochemistry. The Gene Ontology analysis suggested a number of altered biological processes in OTSCCs, including enhancements in phosphate transport, collagen catabolism, I-kappaB kinase/NF-kappaB signaling cascade, extracellular matrix organization and biogenesis, chemotaxis, as well as suppressions of superoxide release, hydrogen peroxide metabolism, cellular response to hydrogen peroxide, keratinization, and keratinocyte differentiation in OTSCCs.

**Conclusion:**

In summary, our study provided a transcriptomic signature for OTSCC that may lead to a diagnosis or screen tool and provide the foundation for further functional validation of these specific candidate genes for OTSCC.

## Background

Head and neck/oral squamous cell carcinoma (HNOSCC) is a complex disease arising in various organs, including oral cavity, tongue, pharynx, and larynx. Tumors from these different sites have distinct clinical presentations and clinical outcomes, and are associated with different risk factors [1] and genetic characteristics [2]. In this study, we focused on the oral tongue squamous cell carcinomas (OTSCC), one of the most common sites for HNOSCCs. The incidence of OTSCC is actually increasing in young and middle age groups [3-5]. OTSCC is significantly more aggressive than other forms of HNOSCCs, with a propensity for rapid local invasion and spread [6].

Cancer cells harbor genetic alterations which are translated into unique expression patterns. These patterns may segregate cancer cells from normal tissue of the same origin and serve as a molecular biomarker. Moreover, expression pattern changes may occur far earlier than clinical disease detection. The identification of such patterns has significant translational values for early detection and diagnosis, as well as for identifying novel therapeutic targets. While several recent studies have attempted to identify expression patterns for HNOSCCs [7-10], to our knowledge, no study has been devoted to identify the unique expression pattern for OTSCC. In this study, we aim to identify the specific transcriptomic/expression patterns that associated with OTSCC.

## Results and discussion

**Figure 1:**
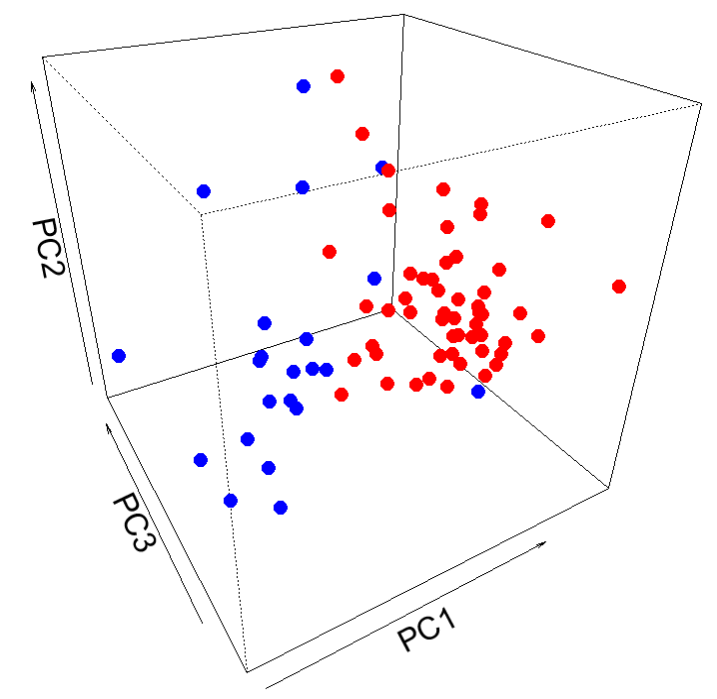
**Principle component analysis**. Global gene expression profiles on 53 OTSCC and 22 matching normal samples were obtained as described in Materials and Methods section. Principle Component (PC) analysis was performed based on the expression profiles of samples. The first 3 PCs were plotted. Red: OTSCC, Blue: normal.

Genome-wide gene expression profiles were obtained on 53 OTSCC samples and 22 normal matching samples. Principal Component Analysis (PCA) was performed based on all the probesets utilized in our microarray analysis. Apparent separation between OTSCC and normal groups was observed with a few outliers (Figure 1). Genes showing statistically significant differences in expression level were identified using RMA and a mixed-effects model as described in the Materials and Methods section. A signature gene set that consists of 35 genes was created using stringent statistical criteria (fold change > 4, and FDR values < 0.0001) (Table 1). Comprehensive lists of genes showing statistically significant upregulations (fold change > 2, and FDR values < 0.01) or downregulations in expression in OTSCC were presented in Supplement Table S1 [see additional file 1] and S2 [see additional file 2], respectively.

**Table 1:** Signature gene sets for OTSCC

	**Symbol**	**Probe ID**	**Gene Name**	**Location**
***Upregulated genes in OTSCC***				
	MMP1	204475_at	matrix metalloproteinase 1	11q22.3
	MMP10	205680_at	matrix metalloproteinase 10	11q22.3
	MMP3	205828_at	matrix metalloproteinase 3	11q22.3
	MMP12	204580_at	matrix metallopeptidase 12	11q22.3
	PTHLH	211756_at	parathyroid hormone-like hormone	12p12.1-p11.2
	INHBA	210511_s_at	inhibin, beta A	7p15-p13
	LAMC2	202267_at	laminin, gamma 2	1q25-q31
	IL8	202859_x_at	interleukin 8	4q13-q21
	KRT17	205157_s_at	keratin 17	17q12-q21
	COL1A2	202404_s_at	collagen, type I, alpha 2	7q22.1
	IFI6	204415_at	interferon, alpha-inducible protein 6	1p35
	ISG15	205483_s_at	ISG15 ubiquitin-like modifier	1p36.33
	PLAU	205479_s_at	plasminogen activator, urokinase	10q24
	GREM1	218468_s_at	gremlin 1	15q13-q15
	MMP9	203936_s_at	matrix metallopeptidase 9	20q11.2-q13.1
	IFI44	214453_s_at	interferon-induced protein 44	1p31.1
	CXCL1	204470_at	chemokine (C-X-C motif) ligand 1	4q21
				
***Downregulated genes in OTSCC***				
	KRT4	213240_s_at	keratin 4	12q12-q13
	MAL	204777_s_at	mal, T-cell differentiation protein	2cen-q13
	CRNN	220090_at	Cornulin	1q21
	SCEL	206884_s_at	Sciellin	13q22
	CRISP3	207802_at	cysteine-rich secretory protein 3	6p12.3
	SPINK5	205185_at	serine protease inhibitor, Kazal type 5	5q32
	CLCA4	220026_at	chloride channel, calcium activated, family member 4	1p31-p22
	ADH1B	209612_s_at 209613_s_at	alcohol dehydrogenase IB (class I), beta polypeptide	4q21-q23
	P11	206605_at	26 serine protease	12q13.1
	TGM3	206004_at	transglutaminase 3	20q11.2
	RHCG	219554_at	Rhesus blood group, C glycoprotein	15q25
	PPP1R3C	204284_at	protein phosphatase 1, regulatory (inhibitor) subunit 3C	10q23-q24
	CEACAM7	206199_at	carcinoembryonic antigen-related cell adhesion molecule 7	19q13.2
	HPGD	203914_x_at	hydroxyprostaglandin dehydrogenase 15-(NAD)	4q34-q35
	CFD	205382_s_at	D component of complement (adipsin)	19p13.3
	ABCA8	204719_at	ATP-binding cassette, sub-family A (ABC1), member 8	17q24
	CLU	222043_at	Clusterin	8p21-p12
	CYP3A5	214235_at	cytochrome P450, family 3, subfamily A, polypeptide 5	7q21.1

* The complete lists of upregulated and downregulated transcripts in OTSCCs were presented in Supplement Table S1 and S2, respectively.

In this study, we identified and validated several interesting potential biomarkers for OTSCC diagnosis. One interesting observation is that 5 members of the Matrix Metalloproteinase (MMP) family (MMP1 MMP3, MMP9, MMP10, and MMP12) are among the genes that most significantly upregulated, which may contribute to the aggressive nature of the OTSCC. MMPs are a large family of proteinases which remodel extracellular matrix (ECM) components and play a significant role in tumor development, survival, invasion and metastasis [11-13]. Several members of the MMP family have been considered to be important biomarkers for diagnosis and prognosis as well as potential therapeutic targets for many types of cancers, including HNOSCC [14]. Our recent study suggested that up regulation of the MMP9 gene is associated with advanced OTSCC and has predictive value for the identification of lymph node metastasis [15]. Here, our data further suggested that MMP9 is one of the biomarkers for the detection of OTSCC. We also observed 2 chemokines (IL8 and CXCL1) to be among the most significantly upregulated genes in OTSCC. The increase in the protein and mRNA of IL8 gene has been suggested as a biomarker for the early detection of oral cancer [16,17]. Our data provided independent validation for this biomarker at the disease tissue level, and suggested that the increase of IL8 molecules (mRNA and protein) is due at least in part to the increased expression of gene in the disease tissues. CXCL1, also known as growth-regulated oncogene 1 (Gro-1), is vital for the survival, progression and invasion of several cancer types [18,19], including oral cancer [20]. Our results here further confirmed the importance of CXCL1 in the tumorgenesis of tongue cancer. Other interesting observations include the up-regulation of KRT17 (associated with invasion and proliferation) and down-regulation of KRT4 (which is associated with squamous cell differentiation), suggesting the potentially distinct roles of KRT genes in tongue SCC development and progression. In addition to those identified biomarkers, our results will also serve as a valuable reference data set for future development and validation of biomarkers for detection, diagnosis and prognosis of tongue cancer.

**Figure 2:**
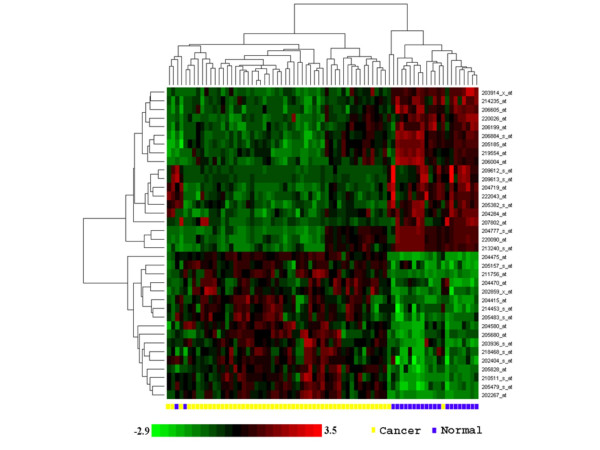
**Classification of OTSCC and matching normal using global gene expression analysis**. A signature gene set of 35 genes was created based on RMA analysis of the expression profiles on 53 OTSCC and 22 matching normal samples. Hierarchical clustering was performed based on this signature gene set. To removed the effect of baseline and scale in the color image, the data from every transcript was standardized (remove mean, divide by standard deviation) before plotting.

To test the utility of this 35-gene signature gene set for classifying OTSCC and normal groups, average linkage hierarchical clustering analysis was performed. As illustrated in Figure 2, our results demonstrated that this 35-gene set provides classification power for OTSCC based on gene expression analyses, which misclassified two cases for each of two groups (a 95% overall accuracy rate).

Our analysis demonstrated that OTSCC can be identified based on the gene expression signature. This finding should provide a foundation for the creation of a specific screen tool for OTSCC. One of the major factors accounting for the poor outcome of OTSCC patients is that a great proportion of oral cancers are diagnosed at advanced stages. Patients diagnosed at an early stage of the disease typically have a better chance for cure and functional outcome. Early detection of tongue cancer lesion will greatly improve patient survival and the quality of life. Current clinical diagnosis and histopathologic examinations are usually based on biopsied material, which requires invasive procedures and surgical techniques. The emerging technology of saliva-based diagnosis may provide an alternative strategy for early diagnosis and screening of the subjects at risk [21]. The markers identified here may be suitable for the saliva-based early diagnosis and screening strategy [17,21]. Additional validation studies will be needed to fully explore this possibility.

To gain a better understanding of the underlying molecular biological processes that dictate the observed expressional pattern alterations in OTSCC development, the Gene Ontology analysis was performed. It was carried out using the GOstats package in the Bioconductor [22] and Gene Ontology Consortium database [23], based on the complete list of 365 differentially expressed transcripts (Supplement Table S1 and S2). Among those 365 genes, 306 were mapped to ENTREZ genes. The gene universe in the analysis consists of 13125 transcripts that were mapped to ENTREZ genes. The Gene Ontology analysis suggested a number of altered biological processes in OTSCC. These include enhancement in phosphate transport, collagen catabolism, I-kappaB kinase/NF-kappaB signaling cascade, extracellular matrix organization and biogenesis, chemotaxis, as well as suppression of superoxide release, hydrogen peroxide metabolism, cellular response to hydrogen peroxide, keratinization, keratinocyte differentiation in OTSCCs (Table 2). The complete lists of enhanced and suppressed biological processes, molecular functions and cellular components in OTSCCs were presented in Supplement Table S3 [see additional file 3] and S4 [see additional file 4], respectively.

**Table 2:** Selected biological processes that altered in OTSCCs *

**GO Name**	**GO ID**	% Change **	**p-value**
***Enhanced***			
Collagen catabolism	GO:0030574	38.9	2.7 E-09
Positive regulation of chemotaxis	GO:0050921	33.3	0.0029
Phosphate transport	GO:0006817	21.7	1.5 E-12
Extracellular matrix organization and biogenesis	GO:0030198	12.9	0.00089
Positive regulation of I-kappaB kinase/NF-kappaB cascade	GO:0043123	8.8	0.00038
			
***Suppressed***			
Superoxide release	GO:0042554	40.0	0.0015
Keratinization	GO:0031424	33.3	1.1 E-05
Hydrogen peroxide metabolism	GO:0042743	25.0	0.0042
Response to hydrogen peroxide	GO:0042542	22.2	0.0053
Keratinocyte differentiation	GO:0030216	21.1	8.0 E-05

* The complete lists of enhanced and suppressed biological processes (BP), molecular functions (MF) and cellular components (CC) in OTSCCs were presented in Supplement Table S3 and S4, respectively.

** The percent of genes exhibiting expressional changes among all genes that constitutes a specific GO entity.

Among the identified alteration in biological activities in OTSCC, the most significantly enhanced are related to the extracellular matrix remodeling (GO:0030574, GO:0030198), I-kappaB kinase/NF-kappaB cascade (GO:0043123) and chemotaxis (GO:0050921), which are known to be related to tumorgenesis and progression of the cancer. One interesting observation is the enhancement in phosphate transport (GO:0006817) in OTSCC. This may be related directly to the enhanced metabolic activity and energy consumption rate in OTSCCs. It has also been suggested that phosphate can act as a signaling molecule on the extracellular signal-regulated kinase (ERK1/2) [24] and adenylate cyclase/cAMP signaling pathways [25], and ultimately affect cell growth. However, the precise role of enhanced phosphate transport in tumorgenesis is largely unclear. The significantly suppressed biological activities, such as superoxide release (GO:0042554), hydrogen peroxide metabolism (GO:0042743), and response to hydrogen peroxide (GO:0042542) are all appeared to be related to the cellular redox state. The effects of redox state in malignancies are somewhat contradictory. In theory, reducing the oxidative stress may prevent DNA degeneration and therefore prevent the development of cancer. However, doing so may also offer increased growth potential to tumor cells and protect them from excess of reactive oxygen species (ROS), which would otherwise lead to apoptosis or necrosis. At the center of this apparent controversy is superoxide dismutase 2 (SOD2), which has been considered as one of the most important antioxidant enzymes. The role of SODs in carcinogenesis has been widely studied but is still rather ambiguous. While the majority of in vitro studies have reported a protective role of SOD2 against tumor progression in cancer cell lines [26-30], including oral cancer cell lines [31], the in vivo studies indicate more complicated roles. Increased SOD2 levels have been observed from esophageal, gastric, brain astrocytic and colorectal carcinomas, and often associated with metastasis and poor prognosis [32-40]. The status of SOD2 in breast cancer is not clear, with some studies showing an increase [41], while others showing a decrease in SOD2 level [42]. Reduction in SOD2 level has been observed in prostatic carcinomas [43,44]. Our microarray results indicated a significant increase in expression of SOD2 gene (probset: 215223_s_at; fold change = 2.37; p value = 0.00014; and probeset: 216841_s_at; fold change = 2.24; p value = 0.000197) in OTSCC. These findings are in agreement with the recent observation in oral cancer [45]. Additional studies will be needed to fully understand the role(s) of redox state and SOD2 in OTSCC.

**Figure 3:**
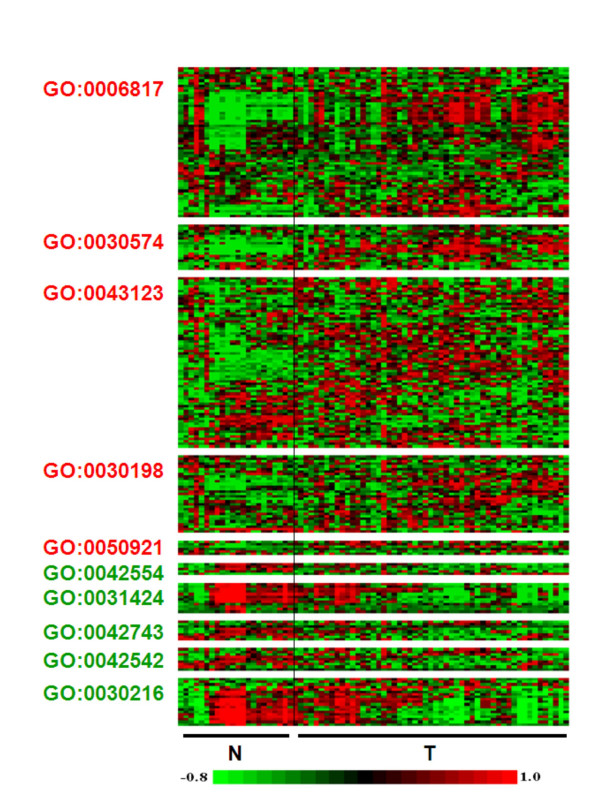
**Expression values of the genes constitute of the altered biological processes in OTSCC**. The altered biological processes in OTSCC were identified by Gene Ontology analysis (Table 2) as described in the Materials and Methods section. The expression values of the genes consisting of the altered biological processes were extracted from the RMA based expression index, and individual heat maps were generated for the identified altered biological processes in OTSCC. To removed the effect of baseline and scale in the color image, the data from every transcript was standardized (remove mean, divide by standard deviation) before plotting. To increase the contrast, values lower than the 10^th ^percentile were replaced with the 10^th ^percentile, and values higher than the 95^th ^percentile were replaced with the 95^th ^percentile.

To visualize the changes in gene expression patterns in relationship with the alteration of biological processes and cellular functions in OTSCC, gene expression heat maps for each identified GO entities were generated based on the microarray results of 53 OTSCC and 22 normal samples (Figure 3). Apparent differences in expression patterns can be observed between OTSCC and normal groups for all the altered GO entities. The summarized statistical values on differential expression for each individual gene of GO:0030574 (collagen catabolism) and GO:0050921 (positive regulation of chemotaxis) are presented in Table 3. The complete expressional analysis for all the altered biological processes identified in Table 2 is presented in Supplement Table S5 [see additional file 5].

**Table 3:** Expression values of genes that constitute the collagen catabolism (GO:0030574) and positive regulation of chemotaxis (GO:0050921) processes in OTSCC.

**GO term**	**entrzID**	**Gene Symbol**	**Fold change**	**FDR level**
***GO:0030574***				
	**4312**	**MMP1**	**57.6**	**0**
	4313	MMP2	1.44	0.109
	**4314**	**MMP3**	**8.43**	**1.01E-08**
	**4316**	**MMP7**	**2.83**	**0.0049**
	4317	MMP8	1.01	0.7963
	**4318**	**MMP9**	**4.08**	**5.85E-05**
	**4319**	**MMP10**	**8.45**	**9.95E-06**
	**4320**	**MMP11**	**2.02**	**0.00085**
	**4322**	**MMP13**	**3.79**	**0.0007**
	4325	MMP16	0.93	0.1117
	4327	MMP19	1.08	0.4404
	5184	PEPD	0.92	0.4602
	5645	PRSS2	0.76	0.0187
	5653	KLK6	0.73	0.4218
	5657	PRTN3	0.96	0.2656
	9508	ADAMTS3	1.05	0.5858
	9509	ADAMTS2	1.19	0.2451
	56547	MMP26	0.94	0.2394
				
***GO:0050921***				
	9353	SLIT2	0.88	0.1700
	566	AZU1	0.95	0.1643
	**3576**	**IL8**	**5.87**	**1.54E-06**
	**6696**	**SPP1**	**3.23**	**0.0027**
	7422	VEGF	1.20	0.4614
	7857	SCG2	1.00	0.9901

**Figure 4:**
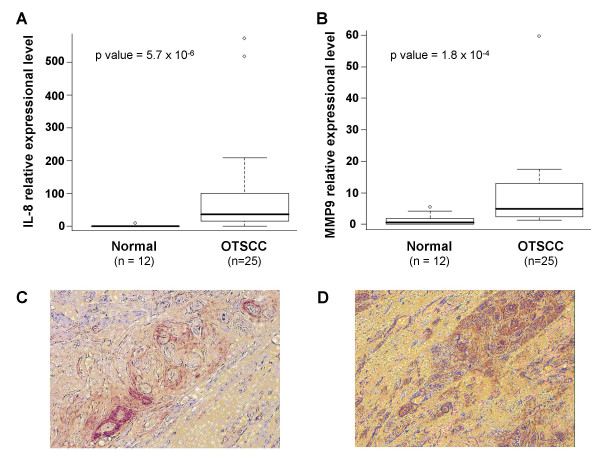
**Elevated expressions of IL8 and MMP9 genes in OTSCC**. The qRT-PCR tests were performed with primer sets specific for IL8 gene (A) and MMP9 gene (B) on 25 OTSCC cases and 12 normal matching samples. The p values (Wilcoxon test) were presented. Immunohistochemistry tests were performed using monoclonal antibody to IL8 and MMP9 and detected using peroxidase-antiperoxidase and diaminebenzadine (DAB) on 10 OTSCC cases. Positive stained SCC cells were observed in 4 cases for IL8 and 8 cases for MMP9. Representative images for IL8 (C) and MMP9 (D) were presented.

Among these differentially expressed genes, some have potential value as diagnosis and prognosis markers, and may be indicative of their respective biological pathways. For example, IL8 is a prototypical chemokine (chemotactic cytokine) and is known for its involvements in the positive regulation of chemotaxis (GO:0050921), which is enhanced in tongue cancers as indicated by our Gene Ontology analysis. IL8 has also been suggested to be a potential biomarker for the early detection of oral cancer [16,17]. The over-expression of the MMP9 gene has been shown to be associated with progression of oral dysplasia to cancer [14]. Our recent study suggested that over-expression of the MMP9 gene is associated with advanced OTSCC and has predictive value for OTSCC lymph node metastasis [15]. The upregulation of MMP9 in OTSCC is also involved in the enhanced collagen catabolism (GO:0030574), as indicated by our Gene Ontology analysis. The qRT-PCR analyze were performed to validate the expressional differences of the IL8 and MMP9 genes between tongue SCCs and normal matching tissues. As shown in Figure 4A and 4B, the differences of both IL8 and MMP9 mRNA levels are statistically significant (p < .05) between OTSCC and normal tissues. The expression of MMP9 and IL8 was further confirmed by immunohistochemistry tests performed using monoclonal antibody to IL8 and MMP9 on 10 OTSCC cases (Figure 4C and 4D). Strong positive stained SCC cells were observed in 4 cases for IL8 and 8 cases for MMP9. The observation of positive staining of IL8 in OTSCC cells confirmed that SCC cells are one of the major sources of IL8 production at the site of oral cancer lesion.

## Conclusion

The HNOSCCs are a diverse group of cancers, that develop from many different anatomic sites and are associated with different risk factors [1] and genetic characteristics [2]. This is the first high-resolution genomic profiling study to our knowledge that has focused on identifying unique expression patterns for tongue cancer (OTSCC). OTSCC is one of the most common types of HNOSCC, and is significantly more aggressive than other forms of HNOSCCs, with a propensity for rapid local invasion and spread [6]. Recent epidemiological studies suggested that the incidence of OTSCC is actually increasing in young and middle age groups [3-5]. In this study, we utilized a relatively large sample size (53 OTSCCs and 22 normal matching samples), which enabled us to capture a precise picture of the genome-wide expression pattern for this disease. It is possible that the genomic portrait of HNOSCC originating from different anatomic sites may be different. More studies will be needed to address this important question.

In summary, we identified the unique expression pattern for OTSCC. Several interesting candidate genes associated with OTSCC were identified. The Gene Ontology analysis indicated that several biological processes and cellular functions are consistently altered in OTSCC. Our results demonstrate the feasibility of utilizing biomarkers discovered by global expression profiling analyses for the detection and diagnosis of OTSCC. In addition, we also provided a valuable reference dataset for future identification and validation of biomarkers for detection, diagnosis and prognosis of OTSCC.

## Methods

### Patients

Microarray data were generated from 26 microdissected OTSCC tissues and 12 matching normal tissue samples. Three additional microarray datasets from 27 OTSCC cases and 10 matching normal control tissues that published previously were either downloaded from GEO database (GSE2280, [46] and GSE3524, [47]) or requested from the authors [7]. Clinical characterizations of these patients are outlined in Table 4. The tumor stages were determined according to the American Joint Committee on Cancer (AJCC) designated classification. This study is approved by Institutional Review Boards at University of California at Los Angeles and at University of Illinois at Chicago.

**Table 4:** Clinical Characterization of the OTSCC Patients*

		OTSCC (n = 53)	Normal (n = 22)
**Age**	Average	57	56
	(Range)	32–82	37–78
**Gender**	Male (%)	73.6	59.1
	Female (%)	26.4	40.9
**Anatomic Site**	Tongue (%)	100	100
**Pathological T Stage**	Stage 4 (%)	56.6	
	Stage 3 (%)	7.5	
	Stage 2 (%)	22.6	
	Stage 1 (%)	13.2	
**Pathological N Stage**	Stage 2 (%)	43.4	
	Stage 1 (%)	7.5	
	Stage 0 (%)	49.1	

* The M stage data is not available.

### Tumor procurement, RNA extraction and microarray hybridization

The OTSCC tissues and their matching normal samples were obtained for this study. These tissues were snap frozen. Cancer tissues containing more than 80% tumor cells based on haematoxylin and eosin (H&E) staining and pathological examination were identified and selectively microdissected by a trained pathologist. The total RNA was isolated using RNeasy Mini kit (Qiagen), and quantified by the RiboGreen RNA Quantitation Reagent (Molecular Probes). A total of 150–200 ng of purified total RNA was amplified by a modified T7 RNA amplification protocol as described previously [15,17]. The Enzo BioArray High Yield RNA Transcript Labeling System (Enzo) was used for labeling the sample prior to hybridization. The biotinylated cRNA (IVT product) was purified using the RNeasy kit (Qiagen). The quantity and purity of the biotinylated cRNA was determined by spectrophotometry and an aliquot of the sample was checked by gel electrophoresis. The samples were hybridized to the Affymetrix Human Genome U133 Plus 2.0 GeneChip arrays according to the Affymetrix protocols. The arrays were scanned with a GeneChip Scanner 3000. The scanned array images were processed with GeneChip Operating software (GCOS), and the CEL files were extracted for further analysis.

### Array data analysis and gene ontology analysis

The CEL files from all datasets (newly generated array data from 26 OTSCCs and 12 matching normals, and additional 27 OTSCCs and 10 normals from published studies [7,46,47]) were imported into the statistical software R 2.4.1 [48] using Bioconductor [49]. The meta-analysis was performed as described [50]. In brief, the Robust Multi-Array Average (RMA) expression measures [51] were computed after background correction and quantile normalization for each microarray dataset. Then, expression values of the overlapping probesets between U133A and U133 Plus 2.0 arrays were extracted. Probeset-level quantile normalization was performed across all samples to make the effect sizes similar among the four datasets. To visualize the overall expression patterns, we performed Principal Component Analysis (PCA) after removing the normal group mean vector separately from each of the four datasets. Finally, for every probeset, a mixed effects model was applied to identify differential expression. For gene *i *in sample *k *of experiment *j*,

$$\begin{array}{ll} y_{ijk}=\mu_{i}+\alpha_{ij}+\beta_{i}x_{ijk}+\varepsilon_{ijk}, & \alpha_{ij}\sim N(0,\delta_{i}),\varepsilon_{ijk}\sim N(0,\sigma_{i})\\ & x_{ijk}=\{\begin{array}{ll} 1, & \text{cancer}\\ 0, & \text{normal} \end{array} \end{array}$$

In the model, the random effect *α *_*ij *_is the laboratory effect, and *β *_*i *_is the first-order cancer effect, which is our major focus in the identification of cancer-associated genes. After obtaining the estimates and the p-values of the *β*_*i*_'s of each probeset, we corrected the p-values for false discovery rate (FDR) [52]. We selected genes at the FDR level of 0.01, and with cancer effect size > 1 (> 2 fold change between cancer and normal samples). Functional analysis of the differentially expressed genes was carried out using the GOstats package in Bioconductor [22] based on the Gene Ontology Consortium database [23].

### Quantitative RT-PCR(qRT-PCR)

The mRNA levels of interleukin-8 (IL8) and matrix metalloproteinases 9 (MMP9) in OTSCCs and normal tissues were further validated using qRT-PCR as previously described [15,17]. The RNA was converted to first strand cDNA using MuLV reverse transcriptase (Applied Biosystems) and the quantitative PCR was performed using iQ SYBR Green Supermix (Bio-Rad) in a BIO-RAD iCycler iQ real-time PCR detection system. The primer sets specific for IL8 (Forward: 5'-GAGGGTTGTGGAGAAGTTTTTG-3', Reverse: 5'-CTGGCATCTTCACTGATTCTTG-3') and for MMP9 (Forward: 5'-GCACGACGTCTTCCAGTACC-3', Reverse: 5'-TCAACTCACTCCGGGAACTC-3') were used. All reactions were performed in triplicate. The melting curve analyses were performed to ensure the specificity of the qRT-PCR reactions. The data analysis was performed using the 2^-deltadelta Ct ^method described previously [53], where beta-actin was used as reference gene. The qRT-PCR based gene expression values between two groups were compared by the nonparametric Wilcoxon test.

### Immunohistochemistry

The expression of IL8 and MMP9 in OTSCCs were further examined using immunohistochemistry tests as previously described [54]. In brief, the OTSCC tissues were processed, embedded, and sectioned at 5 μm. Tissue sections were stained using monoclonal antibody to IL8 (MAB208) (R & D Systems) and MMP9 (ab51203) (Abcam, Inc) and detected using peroxidase-antiperoxidase and diaminebenzadine (DAB) with a Discovery XT automated instrument (Ventana Medical Systems, Inc).

## Authors' contributions

BZ, LM, DW, and XZ conceived the idea for the project and drafted the manuscript. HY, TY, ST, JS, JW and XZ performed the laboratory analyses and conducted statistical analyses. JS and JW provided discussions on clinical relevance. BZ, JS, JW, LM, DW and XZ revised the manuscript. All authors read and approved the final manuscript.

## Supplementary Material

Additional file 1 **Supplement Table S1: Up-regulated transcripts in OTSCC**. The table showing the complete list of the up-regulated transcripts in OTSCC (p value < 0.01; fold increase > 2.0). Click here for file

Additional file 2 **Supplement Table S2: Down-regulated transcripts in OTSCC**. The table showing the complete list of the down-regulated transcripts in OTSCC (p value < 0.01; fold increase < 0.5). Click here for file

Additional file 3 **Supplement Table S3: Enhanced Biological Processes (BP), Molecular Functions (MF) and Cellular Components (CC) in OTSCC**. The table showing the complete list of the enhanced biological processes (BP), molecular functions (MF) and cellular components (CC) in OTSCC (p value < 0.01). Click here for file

Additional file 4 **Supplement Table S4: Suppressed Biological Processes (BP), Molecular Functions (MF) and Cellular Components (CC) in OTSCC**. The table showing the complete list of the suppressed biological processes (BP), molecular functions (MF) and cellular components (CC) in OTSCC (p value < 0.01). Click here for file

Additional file 5 **Supplement Table S5: Expression values of genes that constitute the altered biological processes (listed in **Table 2**) in OTSCC**. The table showing the statistics on expression values of genes that constitute the altered biological processes in OTSCC. Click here for file
