# Supplementary material for: Transcriptomic dissection of tongue squamous cell carcinoma
Source: BMC Genomics. 2008 Feb 6;9:69. doi: 10.1186/1471-2164-9-69 (PMC2262071; doi:10.1186/1471-2164-9-69)
Supplement: Additional file 1 — Supplement Table S1: Up-regulated transcripts in OTSCC. The table showing the complete list of the up-regulated transcripts in OTSCC (p value < 0.01; fold increase > 2.0). [file 1471-2164-9-69-S1.doc]

**Supplement Table S1: Up-regulated transcripts in OTSCC ***

| **Probe ID** | **Gene Symbol** | **Gene Title** | **Chromosomal Location** | **p-value** | **Fold change** |
| --- | --- | --- | --- | --- | --- |
| 204475_at | MMP1 | matrix metallopeptidase 1 (interstitial collagenase) | 11q22.3 | 0 | 57.61528 |
| 205680_at | MMP10 | matrix metallopeptidase 10 (stromelysin 2) | 11q22.3 | 9.95E-06 | 8.448239 |
| 205828_at | MMP3 | matrix metallopeptidase 3 | 11q22.3 | 1.01E-08 | 8.434561 |
| 204580_at | MMP12 | matrix metallopeptidase 12 (macrophage elastase) | 11q22.3 | 7.39E-07 | 7.843088 |
| 211756_at | PTHLH | parathyroid hormone-like hormone | 12p12.1-p11.2 | 2.43E-07 | 7.567635 |
| 210511_s_at | INHBA | inhibin, beta A | 7p15-p13 | 2.95E-07 | 7.007183 |
| 202267_at | LAMC2 | laminin, gamma 2 | 1q25-q31 | 8.83E-08 | 6.874205 |
| 202859_x_at | IL8 | interleukin 8 | 4q13-q21 | 1.54E-06 | 5.866572 |
| 210809_s_at | POSTN | periostin, osteoblast specific factor | 13q13.3 | 0.000127 | 5.386487 |
| 205157_s_at | KRT17 | keratin 17 | 17q12-q21 | 1.70E-05 | 4.822128 |
| 202404_s_at | COL1A2 | collagen, type I, alpha 2 | 7q22.1 | 9.15E-07 | 4.683783 |
| 204415_at | IFI6 | interferon, alpha-inducible protein 6 | 1p35 | 1.24E-06 | 4.661726 |
| 205483_s_at | ISG15 | ISG15 ubiquitin-like modifier | 1p36.33 | 7.50E-06 | 4.615246 |
| 205479_s_at | PLAU | plasminogen activator, urokinase | 10q24 | 1.59E-08 | 4.364222 |
| 218468_s_at | GREM1 | gremlin 1, cysteine knot superfamily, homolog (Xenopus laevis) | 15q13-q15 | 3.66E-05 | 4.190351 |
| 203936_s_at | MMP9 | matrix metallopeptidase 9 | 20q11.2-q13.1 | 5.85E-05 | 4.079094 |
| 214453_s_at | IFI44 | interferon-induced protein 44 | 1p31.1 | 4.96E-07 | 4.057301 |
| 204470_at | CXCL1 | chemokine (C-X-C motif) ligand 1 (melanoma growth stimulating activity, alpha) | 4q21 | 1.44E-05 | 4.009033 |
| 37892_at | COL11A1 | collagen, type XI, alpha 1 | 1p21 | 0.000934 | 4.006326 |
| 221729_at | COL5A2 | collagen, type V, alpha 2 | 2q14-q32 | 5.60E-06 | 3.907361 |
| 211980_at | COL4A1 | collagen, type IV, alpha 1 | 13q34 | 2.62E-07 | 3.893552 |
| 212236_x_at | KRT17 | keratin 17 | 17q12-q21 | 2.66E-05 | 3.887449 |
| 218469_at | GREM1 | gremlin 1, cysteine knot superfamily, homolog (Xenopus laevis) | 15q13-q15 | 6.68E-05 | 3.883477 |
| 205959_at | MMP13 | matrix metallopeptidase 13 (collagenase 3) | 11q22.3 | 0.000712 | 3.791227 |
| 221730_at | COL5A2 | collagen, type V, alpha 2 | 2q14-q32 | 9.62E-05 | 3.781 |
| 213992_at | COL4A6 | collagen, type IV, alpha 6 | Xq22 | 1.99E-06 | 3.757882 |
| 205242_at | CXCL13 | chemokine (C-X-C motif) ligand 13 (B-cell chemoattractant) | 4q21 | 0.000218 | 3.695939 |
| 212364_at | MYO1B | myosin IB | 2q12-q34 | 8.29E-11 | 3.69239 |
| 203413_at | NELL2 | NEL-like 2 (chicken) | 12q13.11-q13.12 | 0.00026 | 3.546042 |
| 201506_at | TGFBI | transforming growth factor, beta-induced, 68kDa | 5q31 | 4.88E-05 | 3.519956 |
| 200790_at | ODC1 | ornithine decarboxylase 1 | 2p25 | 9.04E-05 | 3.500822 |
| 201645_at | TNC | tenascin C (hexabrachion) | 9q33 | 5.69E-05 | 3.493753 |
| 201641_at | BST2 | bone marrow stromal cell antigen 2 | 19p13.2 | 3.85E-06 | 3.425675 |
| 212473_s_at | MICAL2 | microtubule associated monoxygenase, calponin and LIM domain containing 2 | 11p15.3 | 2.14E-06 | 3.356392 |
| 203789_s_at | SEMA3C | sema domain, immunoglobulin domain (Ig), short basic domain, secreted, (semaphorin) 3C | 7q21-q31 | 7.58E-05 | 3.288134 |
| 202411_at | IFI27 | interferon, alpha-inducible protein 27 | 14q32 | 6.98E-05 | 3.276552 |
| 203256_at | CDH3 | cadherin 3, type 1, P-cadherin (placental) | 16q22.1 | 2.17E-07 | 3.264723 |
| 210355_at | PTHLH | parathyroid hormone-like hormone | 12p12.1-p11.2 | 3.75E-06 | 3.262648 |
| 208025_s_at | HMGA2 | high mobility group AT-hook 2 | 12q15 | 0.0001 | 3.237605 |
| 209875_s_at | SPP1 | secreted phosphoprotein 1 | 4q21-q25 | 0.002693 | 3.234513 |
| 206300_s_at | PTHLH | parathyroid hormone-like hormone | 12p12.1-p11.2 | 0.000173 | 3.208346 |
| 203510_at | MET | met proto-oncogene (hepatocyte growth factor receptor) | 7q31 | 2.17E-09 | 3.17755 |
| 202310_s_at | COL1A1 | collagen, type I, alpha 1 | 17q21.33 | 0.000451 | 3.169439 |
| 204948_s_at | FST | follistatin | 5q11.2 | 6.58E-06 | 3.168141 |
| 212488_at | COL5A1 | collagen, type V, alpha 1 | 9q34.2-q34.3 | 0.000308 | 3.06461 |
| 217901_at | DSG2 | Desmoglein 2 | 18q12.1 | 3.97E-05 | 3.049675 |
| 204320_at | COL11A1 | collagen, type XI, alpha 1 | 1p21 | 0.001018 | 3.049474 |
| 201852_x_at | COL3A1 | collagen, type III, alpha 1 | 2q31 | 0.000552 | 3.048682 |
| 203695_s_at | DFNA5 | deafness, autosomal dominant 5 | 7p15 | 2.58E-06 | 3.047308 |
| 210095_s_at | IGFBP3 | insulin-like growth factor binding protein 3 | 7p13-p12 | 3.42E-05 | 3.000012 |
| 212977_at | CMKOR1 | chemokine orphan receptor 1 | 2q37.3 | 0.000739 | 2.991632 |
| 211161_s_at | COL3A1 | collagen, type III, alpha 1 | 2q31 | 0.000391 | 2.98564 |
| 212489_at | COL5A1 | collagen, type V, alpha 1 | 9q34.2-q34.3 | 0.000909 | 2.985532 |
| 204469_at | PTPRZ1 | protein tyrosine phosphatase, receptor-type, Z polypeptide 1 | 7q31.3 | 0.000465 | 2.952378 |
| 211719_x_at | FN1 | fibronectin 1 | 2q34 | 0.004297 | 2.952366 |
| 212365_at | MYO1B | myosin IB | 2q12-q34 | 6.58E-08 | 2.938059 |
| 204439_at | IFI44L | interferon-induced protein 44-like | 1p31.1 | 0.000209 | 2.93317 |
| 219087_at | ASPN | asporin (LRR class 1) | 9q22 | 0.006415 | 2.93226 |
| 202998_s_at | LOXL2 | lysyl oxidase-like 2 | 8p21.3-p21.2 | 6.26E-06 | 2.903303 |
| 203423_at | RBP1 | retinol binding protein 1, cellular | 3q23 | 8.99E-05 | 2.898621 |
| 206513_at | AIM2 | absent in melanoma 2 | 1q22 | 7.82E-05 | 2.886091 |
| 212012_at | PXDN | peroxidasin homolog (Drosophila) | 2p25 | 3.17E-05 | 2.880955 |
| 201250_s_at | SLC2A1 | solute carrier family 2, member 1 | 1p35-p31.3 | 4.62E-06 | 2.878741 |
| 212353_at | SULF1 | sulfatase 1 | 8q13.2-q13.3 | 0.004562 | 2.850256 |
| 203476_at | TPBG | trophoblast glycoprotein | 6q14-q15 | 8.83E-08 | 2.832767 |
| 207345_at | FST | Follistatin | 5q11.2 | 2.62E-05 | 2.83061 |
| 204259_at | MMP7 | matrix metallopeptidase 7 (matrilysin, uterine) | 11q21-q22 | 0.004907 | 2.827885 |
| 203153_at | IFIT1 | interferon-induced protein with tetratricopeptide repeats 1 | 10q25-q26 | 0.001941 | 2.81731 |
| 215076_s_at | COL3A1 | collagen, type III, alpha 1 | 2q31 | 0.000441 | 2.800292 |
| 206569_at | IL24 | interleukin 24 | 1q32 | 0.001346 | 2.796794 |
| 200665_s_at | SPARC | secreted protein, acidic, cysteine-rich (osteonectin) | 5q31.3-q32 | 5.85E-05 | 2.787577 |
| 218847_at | IGF2BP2 | insulin-like growth factor 2 mRNA binding protein 2 | 3q27.2 | 4.91E-06 | 2.781427 |
| 210986_s_at | TPM1 | tropomyosin 1 (alpha) | 15q22.1 | 0.000338 | 2.74112 |
| 213790_at | --- | CDNA FLJ31066 fis, clone HSYRA2001153 | --- | 0.001032 | 2.737036 |
| 219956_at | GALNT6 | UDP-N-acetyl-alpha-D-galactosamine:polypeptide N-acetylgalactosaminyltransferase 6 (GalNAc-T6) | 12q13 | 0.00133 | 2.724982 |
| 212464_s_at | FN1 | fibronectin 1 | 2q34 | 0.00714 | 2.712836 |
| 201579_at | FAT | FAT tumor suppressor homolog 1 (Drosophila) | 4q35 | 5.89E-05 | 2.704397 |
| 201438_at | COL6A3 | collagen, type VI, alpha 3 | 2q37 | 0.000214 | 2.704057 |
| 216442_x_at | FN1 | fibronectin 1 | 2q34 | 0.00551 | 2.702912 |
| 201744_s_at | LUM | Lumican | 12q21.3-q22 | 0.000189 | 2.700033 |
| 210495_x_at | FN1 | fibronectin 1 | 2q34 | 0.005463 | 2.697574 |
| 202270_at | GBP1 | guanylate binding protein 1, interferon-inducible, 67kDa | 1p22.2 | 0.000291 | 2.689541 |
| 209270_at | LAMB3 | laminin, beta 3 | 1q32 | 0.000251 | 2.653953 |
| 200629_at | WARS | tryptophanyl-tRNA synthetase | 14q32.31 | 0.000458 | 2.651657 |
| 212110_at | SLC39A14 | Solute carrier family 39 (zinc transporter), member 14 | 8p21.3 | 1.79E-06 | 2.642716 |
| 203726_s_at | LAMA3 | laminin, alpha 3 | 18q11.2 | 0.000444 | 2.638161 |
| 206343_s_at | NRG1 | neuregulin 1 | 8p21-p12 | 0.000611 | 2.627968 |
| 202269_x_at | GBP1 | guanylate binding protein 1, interferon-inducible, 67kDa | 1p22.2 | 0.000315 | 2.627253 |
| 221898_at | PDPN | podoplanin | 1p36.21 | 3.64E-06 | 2.624543 |
| 209969_s_at | STAT1 | signal transducer and activator of transcription 1, 91kDa | 2q32.2 | 0.000293 | 2.615123 |
| 218986_s_at | FLJ20035 | hypothetical protein FLJ20035 | 4q32.3 | 7.58E-05 | 2.592419 |
| 212013_at | PXDN | peroxidasin homolog (Drosophila) | 2p25 | 0.000267 | 2.566054 |
| 219787_s_at | ECT2 | epithelial cell transforming sequence 2 oncogene | 3q26.1-q26.2 | 3.01E-05 | 2.562727 |
| 213797_at | RSAD2 | Radical S-adenosyl methionine domain containing 2 | 2p25.2 | 0.002365 | 2.538732 |
| 211725_s_at | BID | BH3 interacting domain death agonist | 22q11.1 | 9.62E-08 | 2.531633 |
| 205569_at | LAMP3 | lysosomal-associated membrane protein 3 | 3q26.3-q27 | 0.000897 | 2.528392 |
| 209301_at | CA2 | carbonic anhydrase II | 8q22 | 0.008511 | 2.516831 |
| 221185_s_at | IQCG | IQ motif containing G | 3q29 | 0.001878 | 2.509393 |
| 203562_at | FEZ1 | fasciculation and elongation protein zeta 1 (zygin I) | 11q24.2 | 1.60E-05 | 2.507154 |
| 211964_at | COL4A2 | collagen, type IV, alpha 2 | 13q34 | 0.000214 | 2.503115 |
| 205534_at | PCDH7 | BH-protocadherin (brain-heart) | 4p15 | 0.000624 | 2.48213 |
| 206026_s_at | TNFAIP6 | tumor necrosis factor, alpha-induced protein 6 | 2q23.3 | 5.85E-05 | 2.473472 |
| 200887_s_at | STAT1 | signal transducer and activator of transcription 1, 91kDa | 2q32.2 | 8.32E-06 | 2.46166 |
| 201462_at | SCRN1 | secernin 1 | 7p14.3-p14.1 | 3.71E-07 | 2.461061 |
| 218543_s_at | PARP12 | poly (ADP-ribose) polymerase family, member 12 | 7q34 | 4.74E-07 | 2.44955 |
| 213338_at | TMEM158 | transmembrane protein 158 | 3p21.3 | 3.92E-06 | 2.442903 |
| 218804_at | TMEM16A | transmembrane protein 16A | 11q13.3 | 0.009756 | 2.435487 |
| 217764_s_at | RAB31 | RAB31, member RAS oncogene family | 18p11.3 | 1.01E-06 | 2.433858 |
| 218943_s_at | DDX58 | DEAD (Asp-Glu-Ala-Asp) box polypeptide 58 | 9p12 | 0.000451 | 2.423441 |
| 204686_at | IRS1 | insulin receptor substrate 1 | 2q36 | 6.93E-05 | 2.422471 |
| 213110_s_at | COL4A5 | collagen, type IV, alpha 5 (Alport syndrome) | Xq22 | 0.00011 | 2.415379 |
| 202693_s_at | STK17A | serine/threonine kinase 17a (apoptosis-inducing) | 7p12-p14 | 1.40E-06 | 2.406327 |
| 209546_s_at | APOL1 | apolipoprotein L, 1 | 22q13.1 | 3.38E-06 | 2.400609 |
| 217553_at | MGC87042 | similar to Six transmembrane epithelial antigen of prostate | 7p15.3 | 0.003884 | 2.398106 |
| 213139_at | SNAI2 | snail homolog 2 (Drosophila) | 8q11 | 3.86E-05 | 2.385765 |
| 215223_s_at | SOD2 | superoxide dismutase 2, mitochondrial | 6q25.3 | 0.00014 | 2.374614 |
| 202234_s_at | SLC16A1 | solute carrier family 16, member 1 | 1p12 | 7.60E-05 | 2.369688 |
| 217820_s_at | ENAH | enabled homolog (Drosophila) | 1q42.12 | 1.53E-05 | 2.359696 |
| 217762_s_at | RAB31 | RAB31, member RAS oncogene family | 18p11.3 | 9.06E-06 | 2.355506 |
| 222108_at | AMIGO2 | adhesion molecule with Ig-like domain 2 | 12q13.11 | 0.004372 | 2.315373 |
| 201656_at | ITGA6 | integrin, alpha 6 | 2q31.1 | 1.39E-05 | 2.308611 |
| 203820_s_at | IGF2BP3 | insulin-like growth factor 2 mRNA binding protein 3 | 7p11 | 0.00376 | 2.29516 |
| 202403_s_at | COL1A2 | collagen, type I, alpha 2 | 7q22.1 | 0.00327 | 2.288776 |
| 218888_s_at | NETO2 | neuropilin (NRP) and tolloid (TLL)-like 2 | 16q11 | 2.77E-05 | 2.288419 |
| 209803_s_at | PHLDA2 | pleckstrin homology-like domain, family A, member 2 | 11p15.5 | 0.000163 | 2.284316 |
| 203083_at | THBS2 | thrombospondin 2 | 6q27 | 0.000991 | 2.282711 |
| 217763_s_at | RAB31 | RAB31, member RAS oncogene family | 18p11.3 | 0.000166 | 2.282353 |
| 209900_s_at | SLC16A1 | solute carrier family 16, member 1 | 1p12 | 5.51E-05 | 2.272342 |
| 208892_s_at | DUSP6 | dual specificity phosphatase 6 | 12q22-q23 | 0.004424 | 2.260376 |
| 208691_at | TFRC | transferrin receptor (p90, CD71) | 3q29 | 3.08E-05 | 2.255399 |
| 209955_s_at | FAP | fibroblast activation protein, alpha | 2q23 | 0.002191 | 2.25491 |
| 204992_s_at | PFN2 | profilin 2 | 3q25.1-q25.2 | 0.000716 | 2.248325 |
| 217892_s_at | LIMA1 | LIM domain and actin binding 1 | 12q13 | 2.43E-07 | 2.248122 |
| 201417_at | SOX4 | SRY (sex determining region Y)-box 4 | 6p22.3 | 0.003218 | 2.237252 |
| 216841_s_at | SOD2 | superoxide dismutase 2, mitochondrial | 6q25.3 | 0.000197 | 2.235065 |
| 212590_at | RRAS2 | related RAS viral (r-ras) oncogene homolog 2 | 11p15.2 | 1.37E-06 | 2.22429 |
| 208029_s_at | LAPTM4B | lysosomal associated protein transmembrane 4 beta | 8q22.1 | 8.32E-06 | 2.221307 |
| 202236_s_at | SLC16A1 | solute carrier family 16, member 1 | 1p12 | 6.52E-05 | 2.219167 |
| 201667_at | GJA1 | gap junction protein, alpha 1, 43kDa (connexin 43) | 6q21-q23.2 | 0.000642 | 2.201027 |
| 218618_s_at | FNDC3B | fibronectin type III domain containing 3B | 3q26.31 | 0.00017 | 2.195225 |
| 215177_s_at | ITGA6 | integrin, alpha 6 | 2q31.1 | 0.000537 | 2.193224 |
| 204647_at | HOMER3 | homer homolog 3 (Drosophila) | 19p13.11 | 3.69E-05 | 2.185334 |
| 218717_s_at | LEPREL1 | leprecan-like 1 | 3q28 | 0.009002 | 2.179867 |
| 212190_at | SERPINE2 | serpin peptidase inhibitor, clade E (nexin, plasminogen activator inhibitor type 1), member 2 | 2q33-q35 | 0.007602 | 2.177052 |
| 218193_s_at | GOLT1B | golgi transport 1 homolog B (S. cerevisiae) | 12p12.1 | 0.000125 | 2.173545 |
| 207332_s_at | TFRC | transferrin receptor (p90, CD71) | 3q29 | 0.000125 | 2.170284 |
| 217678_at | SLC7A11 | solute carrier family 7, (cationic amino acid transporter, y+ system) member 11 | 4q28-q32 | 0.002599 | 2.167446 |
| 201976_s_at | MYO10 | myosin X | 5p15.1-p14.3 | 1.45E-06 | 2.164552 |
| 202311_s_at | COL1A1 | collagen, type I, alpha 1 | 17q21.33 | 0.003322 | 2.160899 |
| 217428_s_at | COL10A1 | collagen, type X, alpha 1 | 6q21-q22 | 0.008159 | 2.15613 |
| 201141_at | GPNMB | glycoprotein (transmembrane) nmb | 7p15 | 0.000465 | 2.152528 |
| 213506_at | F2RL1 | coagulation factor II (thrombin) receptor-like 1 | 5q13 | 0.000141 | 2.145534 |
| 214297_at | CSPG4 | Chondroitin sulfate proteoglycan 4 (melanoma-associated) | 15q24.2 | 0.001341 | 2.143674 |
| 214953_s_at | APP | amyloid beta (A4) precursor protein | 21q21.2|21q21.3 | 8.17E-05 | 2.130653 |
| 218542_at | CEP55 | centrosomal protein 55kDa | 10q23.33 | 0.000894 | 2.122924 |
| 205941_s_at | COL10A1 | collagen, type X, alpha 1 | 6q21-q22 | 0.009525 | 2.112299 |
| 219863_at | HERC5 | hect domain and RLD 5 | 4q22.1 | 0.000806 | 2.106345 |
| 210987_x_at | TPM1 | tropomyosin 1 (alpha) | 15q22.1 | 0.002667 | 2.099747 |
| 218644_at | PLEK2 | pleckstrin 2 | 14q23.3 | 0.00097 | 2.091763 |
| 202458_at | PRSS23 | protease, serine, 23 | 11q14.1 | 0.001783 | 2.089092 |
| 220161_s_at | EPB41L4B | erythrocyte membrane protein band 4.1 like 4B | 9q31-q32 | 0.001704 | 2.08734 |
| 211981_at | COL4A1 | collagen, type IV, alpha 1 | 13q34 | 0.002078 | 2.087082 |
| 203921_at | CHST2 | carbohydrate (N-acetylglucosamine-6-O) sulfotransferase 2 | 3q24 | 0.000196 | 2.085496 |
| 204033_at | TRIP13 | thyroid hormone receptor interactor 13 | 5p15.33 | 0.00016 | 2.085301 |
| 221538_s_at | PLXNA1 | plexin A1 | 3q21.3 | 2.27E-05 | 2.084592 |
| 201995_at | EXT1 | exostoses (multiple) 1 | 8q24.11-q24.13 | 1.04E-05 | 2.075684 |
| 214329_x_at | TNFSF10 | tumor necrosis factor (ligand) superfamily, member 10 | 3q26 | 0.009091 | 2.075019 |
| 214039_s_at | LAPTM4B | lysosomal associated protein transmembrane 4 beta | 8q22.1 | 3.75E-06 | 2.070562 |
| 208767_s_at | LAPTM4B | lysosomal associated protein transmembrane 4 beta | 8q22.1 | 0.000223 | 2.0596 |
| 206025_s_at | TNFAIP6 | tumor necrosis factor, alpha-induced protein 6 | 2q23.3 | 0.001578 | 2.054084 |
| 210845_s_at | PLAUR | plasminogen activator, urokinase receptor | 19q13 | 1.87E-05 | 2.052264 |
| 219684_at | RTP4 | receptor transporter protein 4 | 3q27.3 | 0.001069 | 2.046951 |
| 202052_s_at | RAI14 | retinoic acid induced 14 | 5p13.3-p13.2 | 0.000226 | 2.041008 |
| 203744_at | HMGB3 | high-mobility group box 3 | Xq28 | 3.74E-05 | 2.036201 |
| 203819_s_at | IGF2BP3 | insulin-like growth factor 2 mRNA binding protein 3 | 7p11 | 0.006464 | 2.034428 |
| 206632_s_at | APOBEC3B | apolipoprotein B mRNA editing enzyme, catalytic polypeptide-like 3B | 22q13.1-q13.2 | 0.009139 | 2.033656 |
| 203878_s_at | MMP11 | matrix metallopeptidase 11 (stromelysin 3) | 22q11.2|22q11.23 | 0.000847 | 2.027893 |
| 201505_at | LAMB1 | laminin, beta 1 | 7q22 | 0.002788 | 2.025268 |
| 213294_at | --- | Full-length cDNA clone CS0DK002YF13 of HeLa cells Cot 25-normalized of Homo sapiens (human) | --- | 0.000125 | 2.024 |
| 201300_s_at | PRNP | prion protein (p27-30) | 20p13 | 1.79E-06 | 2.023271 |
| 219763_at | DENND1A | DENN/MADD domain containing 1A | 9q33.2 | 0.000173 | 2.021333 |
| 200755_s_at | CALU | Calumenin | 7q32 | 6.58E-06 | 2.019635 |
| 209576_at | GNAI1 | guanine nucleotide binding protein (G protein), alpha inhibiting activity polypeptide 1 | 7q21 | 1.13E-05 | 2.007893 |
| 202351_at | ITGAV | integrin, alpha V | 2q31-q32 | 4.40E-05 | 2.005868 |
| 202644_s_at | TNFAIP3 | tumor necrosis factor, alpha-induced protein 3 | 6q23 | 0.000271 | 2.003325 |
| 201920_at | SLC20A1 | solute carrier family 20, member 1 | 2q11-q14 | 8.13E-05 | 2.001347 |
| 205660_at | OASL | 2'-5'-oligoadenylate synthetase-like | 12q24.2 | 0.006281 | 2.001053 |
| 202600_s_at | NRIP1 | nuclear receptor interacting protein 1 | 21q11.2 | 0.002661 | 2.000704 |

* p value < 0.01; fold increase > 2.0
