# Supplementary material for: Transcriptomic dissection of tongue squamous cell carcinoma
Source: BMC Genomics. 2008 Feb 6;9:69. doi: 10.1186/1471-2164-9-69 (PMC2262071; doi:10.1186/1471-2164-9-69)
Supplement: Additional file 2 — Supplement Table S2: Down-regulated transcripts in OTSCC. The table showing the complete list of the down-regulated transcripts in OTSCC (p value < 0.01; fold increase < 0.5). [file 1471-2164-9-69-S2.doc]

**Supplement Table S2: Down-regulated transcripts in OTSCC ***

| **Probe ID** | **Gene Symbol** | **Gene Title** | **Chromosomal Location** | **p-value** | **Fold change** |
| --- | --- | --- | --- | --- | --- |
| 213240_s_at | KRT4 | keratin 4 | 12q12-q13 | 1.67E-06 | 0.050358 |
| 204777_s_at | MAL | mal, T-cell differentiation protein | 2cen-q13 | 3.97E-07 | 0.0512 |
| 220090_at | CRNN | cornulin | 1q21 | 1.91E-06 | 0.053086 |
| 207935_s_at | KRT13 | keratin 13 | 17q12-q21.2 | 0.000509 | 0.08558 |
| 206884_s_at | SCEL | sciellin | 13q22 | 4.63E-07 | 0.110991 |
| 207802_at | CRISP3 | cysteine-rich secretory protein 3 | 6p12.3 | 6.51E-05 | 0.120375 |
| 218990_s_at | SPRR3 | small proline-rich protein 3 | 1q21-q22 | 0.002721 | 0.135765 |
| 205185_at | SPINK5 | serine peptidase inhibitor, Kazal type 5 | 5q32 | 7.68E-06 | 0.142829 |
| 220026_at | CLCA4 | chloride channel, calcium activated, family member 4 | 1p31-p22 | 6.75E-06 | 0.152554 |
| 209612_s_at | ADH1B | alcohol dehydrogenase IB (class I), beta polypeptide | 4q21-q23 | 1.59E-08 | 0.15979 |
| 206605_at | P11 | 26 serine protease | 12q13.1 | 9.62E-08 | 0.163667 |
| 206004_at | TGM3 | transglutaminase 3 | 20q11.2 | 1.61E-05 | 0.172884 |
| 219554_at | RHCG | Rh family, C glycoprotein | 15q25 | 9.96E-05 | 0.190647 |
| 204284_at | PPP1R3C | protein phosphatase 1, regulatory (inhibitor) subunit 3C | 10q23-q24 | 8.37E-06 | 0.208755 |
| 206199_at | CEACAM7 | carcinoembryonic antigen-related cell adhesion molecule 7 | 19q13.2 | 5.59E-06 | 0.214557 |
| 204737_s_at | MYH6 /// MYH7 | myosin, heavy polypeptide 6, cardiac muscle, alpha /// myosin, heavy polypeptide 7, cardiac muscle, beta | 14q12 | 0.005654 | 0.217554 |
| 209613_s_at | ADH1B | alcohol dehydrogenase IB (class I), beta polypeptide | 4q21-q23 | 8.00E-07 | 0.222245 |
| 203914_x_at | HPGD | hydroxyprostaglandin dehydrogenase 15-(NAD) | 4q34-q35 | 9.16E-08 | 0.226633 |
| 209687_at | CXCL12 | chemokine (C-X-C motif) ligand 12 (stromal cell-derived factor 1) | 10q11.1 | 0.000785 | 0.228046 |
| 205382_s_at | CFD | complement factor D (adipsin) | 19p13.3 | 1.40E-06 | 0.228892 |
| 213371_at | LDB3 | LIM domain binding 3 | 10q22.3-q23.2 | 0.001125 | 0.230323 |
| 204719_at | ABCA8 | ATP-binding cassette, sub-family A (ABC1), member 8 | 17q24 | 5.16E-06 | 0.231521 |
| 222043_at | CLU | clusterin | 8p21-p12 | 1.06E-05 | 0.239177 |
| 220620_at | C1orf42 | chromosome 1 open reading frame 42 | 1q21 | 0.000595 | 0.243608 |
| 214235_at | CYP3A5 | cytochrome P450, family 3, subfamily A, polypeptide 5 | 7q21.1 | 4.63E-07 | 0.246038 |
| 211737_x_at | PTN | pleiotrophin (heparin binding growth factor 8, neurite growth-promoting factor 1) | 7q33-q34 | 4.96E-07 | 0.251359 |
| 209763_at | CHRDL1 | chordin-like 1 | Xq22.3 | 2.31E-05 | 0.25263 |
| 211597_s_at | HOP | homeodomain-only protein | 4q11-q12 | 0.000109 | 0.262894 |
| 201325_s_at | EMP1 | epithelial membrane protein 1 | 12p12.3 | 5.37E-07 | 0.266213 |
| 211548_s_at | HPGD | hydroxyprostaglandin dehydrogenase 15-(NAD) | 4q34-q35 | 3.80E-07 | 0.267729 |
| 201884_at | CEACAM5 | carcinoembryonic antigen-related cell adhesion molecule 5 | 19q13.1-q13.2 | 0.000769 | 0.270884 |
| 214063_s_at | TF | Transferring | 3q22.1 | 0.000785 | 0.271463 |
| 201324_at | EMP1 | epithelial membrane protein 1 | 12p12.3 | 2.62E-07 | 0.276271 |
| 212510_at | GPD1L | glycerol-3-phosphate dehydrogenase 1-like | 3p22.3 | 7.43E-08 | 0.280631 |
| 203961_at | NEBL | Nebulette | 10p12 | 1.81E-06 | 0.284048 |
| 214399_s_at | KRT8 | Keratin 8 | 12q13 | 5.59E-06 | 0.29015 |
| 210096_at | CYP4B1 | cytochrome P450, family 4, subfamily B, polypeptide 1 | 1p34-p12 | 4.20E-05 | 0.294115 |
| 204570_at | COX7A1 | cytochrome c oxidase subunit VIIa polypeptide 1 | 19q13.1 | 0.000342 | 0.300063 |
| 220431_at | TMPRSS11E | transmembrane protease, serine 11E | 4q13.2 | 0.000281 | 0.303501 |
| 204753_s_at | HLF | hepatic leukemia factor | 17q22 | 2.15E-07 | 0.304885 |
| 209365_s_at | ECM1 | extracellular matrix protein 1 | 1q21 | 8.24E-06 | 0.30796 |
| 207761_s_at | METTL7A | methyltransferase like 7A | 12q13.13 | 0.000162 | 0.308639 |
| 204483_at | ENO3 | enolase 3 (beta, muscle) | 17pter-p11 | 0.006533 | 0.312551 |
| 206023_at | NMU | neuromedin U | 4q12 | 6.31E-05 | 0.314672 |
| 209242_at | PEG3 | paternally expressed 3 | 19q13.4 | 0.000818 | 0.320181 |
| 215704_at | FLG | Filaggrin | 1q21.3 | 0.00116 | 0.321361 |
| 211026_s_at | MGLL | monoglyceride lipase | 3q21.3 | 3.80E-07 | 0.321579 |
| 207175_at | ADIPOQ | adiponectin, C1Q and collagen domain containing | 3q27 | 0.00035 | 0.322687 |
| 206008_at | TGM1 | transglutaminase 1 (K polypeptide epidermal type I, protein-glutamine-gamma-glutamyltransferase) | 14q11.2 | 0.003627 | 0.322892 |
| 203296_s_at | ATP1A2 | ATPase, Na+/K+ transporting, alpha 2 (+) polypeptide | 1q21-q23 | 0.006235 | 0.323235 |
| 207602_at | TMPRSS11D | transmembrane protease, serine 11D | 4q13.2 | 0.002622 | 0.324075 |
| 213451_x_at | TNXB | tenascin XB | 6p21.3 | 1.32E-07 | 0.324392 |
| 203585_at | ZNF185 | zinc finger protein 185 (LIM domain) | Xq28 | 6.93E-05 | 0.325255 |
| 207206_s_at | ALOX12 | arachidonate 12-lipoxygenase | 17p13.1 | 5.88E-05 | 0.325564 |
| 206227_at | CILP | cartilage intermediate layer protein, nucleotide pyrophosphohydrolase | 15q22 | 2.08E-05 | 0.326899 |
| 209894_at | LEPR | leptin receptor | 1p31 | 0.000161 | 0.32778 |
| 209291_at | ID4 | inhibitor of DNA binding 4, dominant negative helix-loop-helix protein | 6p22-p21 | 7.00E-08 | 0.327922 |
| 201348_at | GPX3 | glutathione peroxidase 3 (plasma) | 5q23 | 0.000175 | 0.330296 |
| 204734_at | KRT15 | keratin 15 | 17q21.2 | 0.006753 | 0.330673 |
| 204939_s_at | PLN | Phospholamban | 6q22.1 | 0.002579 | 0.335346 |
| 205765_at | CYP3A5 | cytochrome P450, family 3, subfamily A, polypeptide 5 | 7q21.1 | 4.69E-07 | 0.335846 |
| 203675_at | NUCB2 | nucleobindin 2 | 11p15.1-p14 | 3.86E-05 | 0.337262 |
| 203453_at | SCNN1A | sodium channel, nonvoltage-gated 1 alpha | 12p13 | 0.005432 | 0.337976 |
| 213071_at | DPT | Dermatopontin | 1q12-q23 | 5.75E-06 | 0.339887 |
| 212657_s_at | IL1RN | interleukin 1 receptor antagonist | 2q14.2 | 0.001968 | 0.340381 |
| 212276_at | LPIN1 | lipin 1 | 2p25.1 | 2.62E-07 | 0.340837 |
| 214536_at | SLURP1 | secreted LY6/PLAUR domain containing 1 | 8q24.3 | 0.000608 | 0.343133 |
| 206093_x_at | TNXB | tenascin XB | 6p21.3 | 3.35E-10 | 0.345674 |
| 216333_x_at | TNXB | tenascin XB | 6p21.3 | 9.16E-08 | 0.346391 |
| 203400_s_at | TF | Transferring | 3q22.1 | 0.005654 | 0.347735 |
| 202037_s_at | SFRP1 | secreted frizzled-related protein 1 | 8p12-p11.1 | 0.00325 | 0.347869 |
| 221524_s_at | RRAGD | Ras-related GTP binding D | 6q15-q16 | 0.000401 | 0.350638 |
| 204755_x_at | HLF | hepatic leukemia factor | 17q22 | 1.59E-08 | 0.354832 |
| 203571_s_at | C10orf116 | chromosome 10 open reading frame 116 | 10q23.2 | 3.50E-07 | 0.358415 |
| 207655_s_at | BLNK | B-cell linker | 10q23.2-q23.33 | 6.11E-05 | 0.35957 |
| 205783_at | KLK13 | kallikrein 13 | 19q13.3-q13.4 | 0.007153 | 0.360153 |
| 209498_at | CEACAM1 | carcinoembryonic antigen-related cell adhesion molecule 1 (biliary glycoprotein) | 19q13.2 | 0.000302 | 0.360615 |
| 213068_at | DPT | Dermatopontin | 1q12-q23 | 0.001093 | 0.366478 |
| 205554_s_at | DNASE1L3 | deoxyribonuclease I-like 3 | 3p21.1-3p14.3 | 4.16E-06 | 0.366738 |
| 203498_at | DSCR1L1 | Down syndrome critical region gene 1-like 1 | 6p12.3 | 0.000584 | 0.368409 |
| 203407_at | PPL | Periplakin | 16p13.3 | 0.003842 | 0.370003 |
| 212274_at | LPIN1 | lipin 1 | 2p25.1 | 2.15E-07 | 0.370722 |
| 204865_at | CA3 | carbonic anhydrase III, muscle specific | 8q13-q22 | 0.002947 | 0.371619 |
| 203962_s_at | NEBL | Nebulette | 10p12 | 4.50E-05 | 0.372427 |
| 205200_at | CLEC3B | C-type lectin domain family 3, member B | 3p22-p21.3 | 2.61E-07 | 0.376764 |
| 208126_s_at | CYP2C18 | cytochrome P450, family 2, subfamily C, polypeptide 18 | 10q24 | 0.0019 | 0.376986 |
| 217508_s_at | C18orf25 | chromosome 18 open reading frame 25 | 18q21.1 | 3.64E-06 | 0.378993 |
| 213572_s_at | SERPINB1 | serpin peptidase inhibitor, clade B (ovalbumin), member 1 | 6p25 | 0.001294 | 0.382727 |
| 205623_at | ALDH3A1 | aldehyde dehydrogenase 3 family, memberA1 | 17p11.2 | 0.004637 | 0.385255 |
| 221523_s_at | RRAGD | Ras-related GTP binding D | 6q15-q16 | 0.000558 | 0.388173 |
| 91826_at | EPS8L1 | EPS8-like 1 | 19q13.42 | 0.000229 | 0.388348 |
| 211726_s_at | FMO2 | flavin containing monooxygenase 2 (non-functional) | 1q23-q25 | 3.63E-05 | 0.388934 |
| 221665_s_at | EPS8L1 | EPS8-like 1 | 19q13.42 | 4.65E-05 | 0.393126 |
| 218807_at | VAV3 | vav 3 oncogene | 1p13.3 | 0.000403 | 0.393999 |
| 216265_x_at | MYH7 | myosin, heavy polypeptide 7, cardiac muscle, beta | 14q12 | 0.006686 | 0.395883 |
| 215125_s_at | UGT1A10 /// UGT1A8 /// UGT1A7 /// UGT1A6 /// UGT1A5 /// UGT1A9 /// UGT1A4 /// UGT1A1 /// UGT1A3 | UDP glucuronosyltransferase 1 family, polypeptide A10 /// UDP glucuronosyltransferase 1 family, polypeptide A8 /// UDP glucuronosyltransferase 1 family, polypeptide A7 /// UDP glucuronosyltransferase 1 family, polypeptide A6 /// UDP glucuronosyltransferase 1 family, polypeptide A5 /// UDP glucuronosyltransferase 1 family, polypeptide A9 /// UDP glucuronosyltransferase 1 family, polypeptide A4 /// UDP glucuronosyltransferase 1 family, polypeptide A1 /// UDP glucuronosyltransferase 1 family, polypeptide A3 | 2q37 | 0.00983 | 0.397897 |
| 208650_s_at | CD24 | CD24 molecule | 6q21 | 0.003906 | 0.399096 |
| 219476_at | C1orf116 | chromosome 1 open reading frame 116 | 1q32.1 | 0.000269 | 0.399859 |
| 202746_at | ITM2A | integral membrane protein 2A | Xq13.3-Xq21.2 | 0.002512 | 0.403237 |
| 209466_x_at | PTN | pleiotrophin (heparin binding growth factor 8, neurite growth-promoting factor 1) | 7q33-q34 | 9.06E-06 | 0.403828 |
| 205637_s_at | SH3GL3 | SH3-domain GRB2-like 3 | 15q24 | 1.55E-05 | 0.409139 |
| 205694_at | TYRP1 | tyrosinase-related protein 1 | 9p23 | 0.007376 | 0.41277 |
| 41644_at | SASH1 | SAM and SH3 domain containing 1 | 6q24.3 | 4.71E-08 | 0.412895 |
| 212268_at | SERPINB1 | serpin peptidase inhibitor, clade B (ovalbumin), member 1 | 6p25 | 0.005308 | 0.413557 |
| 209457_at | DUSP5 | dual specificity phosphatase 5 | 10q25 | 1.36E-05 | 0.416402 |
| 201497_x_at | MYH11 | myosin, heavy polypeptide 11, smooth muscle | 16p13.11 | 0.000849 | 0.417634 |
| 220197_at | ATP6V0A4 | ATPase, H+ transporting, lysosomal V0 subunit a4 | 7q33-q34 | 4.42E-05 | 0.419468 |
| 218552_at | ECHDC2 | enoyl Coenzyme A hydratase domain containing 2 | 1p32.3 | 2.15E-07 | 0.420859 |
| 213174_at | TTC9 | tetratricopeptide repeat domain 9 | 14q24.2 | 1.44E-05 | 0.420986 |
| 203845_at | PCAF | p300/CBP-associated factor | 3p24 | 1.16E-06 | 0.421835 |
| 204041_at | MAOB | monoamine oxidase B | Xp11.23 | 3.19E-07 | 0.42237 |
| 213236_at | SASH1 | SAM and SH3 domain containing 1 | 6q24.3 | 1.39E-06 | 0.424117 |
| 218779_x_at | EPS8L1 | EPS8-like 1 | 19q13.42 | 0.000166 | 0.425337 |
| 204955_at | SRPX | sushi-repeat-containing protein, X-linked | Xp21.1 | 0.002029 | 0.425847 |
| 207878_at | KRT76 | keratin 76 | 12q13.13 | 0.002108 | 0.427366 |
| 206877_at | MXD1 | MAX dimerization protein 1 | 2p13-p12 | 0.00014 | 0.427445 |
| 219597_s_at | DUOX1 | dual oxidase 1 | 15q15.3 | 0.001368 | 0.428157 |
| 219529_at | CLIC3 /// RABEP1 | chloride intracellular channel 3 /// rabaptin, RAB GTPase binding effector protein 1 | 9q34.3 /// 17p13.2 | 0.002655 | 0.428434 |
| 206482_at | PTK6 | PTK6 protein tyrosine kinase 6 | 20q13.3 | 0.000219 | 0.431592 |
| 218677_at | S100A14 | S100 calcium binding protein A14 | 1q21.3 | 0.001311 | 0.431614 |
| 218858_at | DEPDC6 | DEP domain containing 6 | 8q24.12 | 0.001069 | 0.435499 |
| 201535_at | UBL3 | ubiquitin-like 3 | 13q12-q13 | 4.46E-05 | 0.439798 |
| 205987_at | CD1C | CD1c molecule | 1q22-q23 | 8.55E-09 | 0.440442 |
| 214576_at | KRT36 | keratin 36 | 17q12-q21 | 0.003435 | 0.441686 |
| 214624_at | UPK1A | uroplakin 1A | 19q13.13 | 0.000107 | 0.442477 |
| 214091_s_at | GPX3 | glutathione peroxidase 3 (plasma) | 5q23 | 0.001018 | 0.445437 |
| 203697_at | FRZB | frizzled-related protein | 2qter | 0.000927 | 0.44596 |
| 221748_s_at | TNS1 | tensin 1 /// tensin 1 | 2q35-q36 | 0.00376 | 0.446432 |
| 221675_s_at | CHPT1 | choline phosphotransferase 1 | 12q | 0.001544 | 0.448277 |
| 221655_x_at | EPS8L1 | EPS8-like 1 | 19q13.42 | 0.000196 | 0.448675 |
| 204069_at | MEIS1 | Meis1, myeloid ecotropic viral integration site 1 homolog (mouse) | 2p14-p13 | 2.14E-06 | 0.449879 |
| 202054_s_at | ALDH3A2 | aldehyde dehydrogenase 3 family, member A2 | 17p11.2 | 2.07E-05 | 0.450954 |
| 205392_s_at | CCL14 /// CCL15 | chemokine (C-C motif) ligand 14 /// chemokine (C-C motif) ligand 15 | 17q11.2 | 2.43E-07 | 0.452549 |
| 213421_x_at | PRSS3 | protease, serine, 3 (mesotrypsin) | 9p11.2 | 0.007153 | 0.453732 |
| 204532_x_at | UGT1A4 | UDP glucuronosyltransferase 1 family, polypeptide A4 | 2q37 | 0.003936 | 0.453969 |
| 204393_s_at | ACPP | acid phosphatase, prostate | 3q21-q23 | 0.000308 | 0.454347 |
| 203662_s_at | TMOD1 | tropomodulin 1 | 9q22.3 | 0.002115 | 0.455903 |
| 217031_at | KRT84 | keratin 84 | 12q13 | 0.003636 | 0.456379 |
| 38158_at | ESPL1 | extra spindle poles like 1 (S. cerevisiae) | 12q | 8.10E-07 | 0.4568 |
| 208335_s_at | DARC | Duffy blood group, chemokine receptor | 1q21-q22 | 0.000383 | 0.457489 |
| 205478_at | PPP1R1A | protein phosphatase 1, regulatory (inhibitor) subunit 1A | 12q13.2 | 0.000237 | 0.458059 |
| 219681_s_at | RAB11FIP1 | RAB11 family interacting protein 1 (class I) | 8p11.22 | 0.00783 | 0.458648 |
| 206094_x_at | UGT1A6 | UDP glucuronosyltransferase 1 family, polypeptide A6 | 2q37 | 0.005229 | 0.458701 |
| 200795_at | SPARCL1 | SPARC-like 1 (mast9, hevin) | 4q22.1 | 0.007443 | 0.460534 |
| 209667_at | CES2 | carboxylesterase 2 (intestine, liver) | 16q22.1 | 0.000764 | 0.462514 |
| 221667_s_at | HSPB8 | heat shock 22kDa protein 8 | 12q24.23 | 0.000688 | 0.462938 |
| 220289_s_at | AIM1L | absent in melanoma 1-like | 1p36.11 | 0.000555 | 0.463284 |
| 203913_s_at | HPGD | hydroxyprostaglandin dehydrogenase 15-(NAD) | 4q34-q35 | 0.000447 | 0.464508 |
| 213509_x_at | CES2 | carboxylesterase 2 (intestine, liver) | 16q22.1 | 0.00013 | 0.466604 |
| 207126_x_at | UGT1A4 | UDP glucuronosyltransferase 1 family, polypeptide A4 | 2q37 | 0.006602 | 0.466616 |
| 209772_s_at | CD24 | CD24 molecule | 6q21 | 0.001042 | 0.467147 |
| 210505_at | ADH7 | alcohol dehydrogenase 7 (class IV), mu or sigma polypeptide | 4q23-q24 | 0.002314 | 0.467568 |
| 218885_s_at | GALNT12 | UDP-N-acetyl-alpha-D-galactosamine:polypeptide N-acetylgalactosaminyltransferase 12 (GalNAc-T12) | 9q22.33 | 0.001468 | 0.470117 |
| 211959_at | IGFBP5 | insulin-like growth factor binding protein 5 | 2q33-q36 | 0.009079 | 0.471204 |
| 220149_at | FLJ22671 | hypothetical protein FLJ22671 | 2q37.3 | 2.00E-07 | 0.473196 |
| 209373_at | MALL | mal, T-cell differentiation protein-like | 2q13 | 0.005382 | 0.476566 |
| 212593_s_at | PDCD4 | programmed cell death 4 (neoplastic transformation inhibitor) | 10q24 | 3.97E-07 | 0.478764 |
| 49452_at | ACACB | acetyl-Coenzyme A carboxylase beta | 12q24.11 | 0.000167 | 0.479273 |
| 205081_at | CRIP1 /// GALK2 | cysteine-rich protein 1 (intestinal) /// galactokinase 2 | 14q32.33 /// 15q21.1 | 0.008207 | 0.48305 |
| 209318_x_at | PLAGL1 | pleiomorphic adenoma gene-like 1 | 6q24-q25 | 0.001932 | 0.483708 |
| 213895_at | EMP1 | epithelial membrane protein 1 | 12p12.3 | 6.58E-06 | 0.484937 |
| 212865_s_at | COL14A1 | collagen, type XIV, alpha 1 (undulin) | 8q23 | 0.005555 | 0.485316 |
| 209074_s_at | FAM107A | family with sequence similarity 107, member A | 3p21.1 | 1.07E-06 | 0.486231 |
| 204731_at | TGFBR3 | transforming growth factor, beta receptor III (betaglycan, 300kDa) | 1p33-p32 | 0.001544 | 0.486642 |
| 219213_at | JAM2 | junctional adhesion molecule 2 | 21q21.2 | 0.001522 | 0.486869 |
| 212543_at | AIM1 | absent in melanoma 1 | 6q21 | 0.000536 | 0.487377 |
| 213929_at | --- | Homo sapiens, Similar to likely ortholog of yeast ARV1, clone IMAGE:4733238, mRNA | --- | 1.33E-05 | 0.487477 |
| 204168_at | MGST2 | microsomal glutathione S-transferase 2 | 4q28.3 | 2.87E-07 | 0.488852 |
| 209975_at | CYP2E1 | cytochrome P450, family 2, subfamily E, polypeptide 1 | 10q24.3-qter | 0.001677 | 0.488971 |
| 207002_s_at | PLAGL1 | pleiomorphic adenoma gene-like 1 | 6q24-q25 | 0.001119 | 0.491662 |
| 214433_s_at | SELENBP1 | selenium binding protein 1 | 1q21-q22 | 3.41E-05 | 0.492055 |
| 208796_s_at | CCNG1 | cyclin G1 | 5q32-q34 | 0.000379 | 0.493948 |
| 210944_s_at | CAPN3 | calpain 3, (p94) | 15q15.1-q21.1 | 0.008032 | 0.49637 |
| 201201_at | CSTB | cystatin B (stefin B) | 21q22.3 | 0.003628 | 0.496626 |
| 204817_at | ESPL1 | extra spindle poles like 1 (S. cerevisiae) | 12q | 3.74E-06 | 0.496826 |
| 214168_s_at | TJP1 | tight junction protein 1 (zona occludens 1) | 15q13 | 8.04E-05 | 0.497113 |
| 221404_at | IL1F6 | interleukin 1 family, member 6 (epsilon) | 2q12-q14.1 | 1.81E-06 | 0.497465 |

* p value < 0.01; fold increase < 0.5
