# Supplementary material for: Transcriptomic dissection of tongue squamous cell carcinoma
Source: BMC Genomics. 2008 Feb 6;9:69. doi: 10.1186/1471-2164-9-69 (PMC2262071; doi:10.1186/1471-2164-9-69)
Supplement: Additional file 3 — Supplement Table S3: Enhanced Biological Processes (BP), Molecular Functions (MF) and Cellular Components (CC) in OTSCC. The table showing the complete list of the enhanced biological processes (BP), molecular functions (MF) and cellular components (CC) in OTSCC (p value < 0.01). [file 1471-2164-9-69-S3.doc]

**Supplement Table S3: Enhanced Biological Processes (BP), Molecular Functions (MF) and Cellular Components (CC) in OTSCC ***

| **GO ID** | **Sig Genes** | **Genes in GO** | **Proportion of Change** | **P value** | **GO category** | **GO Term** |
| --- | --- | --- | --- | --- | --- | --- |
| GO:0046880 | 2 | 4 | 0.5 | 0.001172 | BP | [regulation of follicle-stimulating hormone secretion](http://www.godatabase.org/cgi-bin/amigo/go.cgi?view=details&search_constraint=terms&depth=0&query=GO:0046880) |
| GO:0046882 | 2 | 4 | 0.5 | 0.001172 | BP | [negative regulation of follicle-stimulating hormone secretion](http://www.godatabase.org/cgi-bin/amigo/go.cgi?view=details&search_constraint=terms&depth=0&query=GO:0046882) |
| GO:0046884 | 2 | 4 | 0.5 | 0.001172 | BP | [follicle-stimulating hormone secretion](http://www.godatabase.org/cgi-bin/amigo/go.cgi?view=details&search_constraint=terms&depth=0&query=GO:0046884) |
| GO:0046888 | 2 | 4 | 0.5 | 0.001172 | BP | [negative regulation of hormone secretion](http://www.godatabase.org/cgi-bin/amigo/go.cgi?view=details&search_constraint=terms&depth=0&query=GO:0046888) |
| GO:0051048 | 2 | 5 | 0.4 | 0.001935 | BP | [negative regulation of secretion](http://www.godatabase.org/cgi-bin/amigo/go.cgi?view=details&search_constraint=terms&depth=0&query=GO:0051048) |
| GO:0050926 | 2 | 5 | 0.4 | 0.001935 | BP | [regulation of positive chemotaxis](http://www.godatabase.org/cgi-bin/amigo/go.cgi?view=details&search_constraint=terms&depth=0&query=GO:0050926) |
| GO:0050927 | 2 | 5 | 0.4 | 0.001935 | BP | [positive regulation of positive chemotaxis](http://www.godatabase.org/cgi-bin/amigo/go.cgi?view=details&search_constraint=terms&depth=0&query=GO:0050927) |
| GO:0050918 | 2 | 5 | 0.4 | 0.001935 | BP | [positive chemotaxis](http://www.godatabase.org/cgi-bin/amigo/go.cgi?view=details&search_constraint=terms&depth=0&query=GO:0050918) |
| GO:0050930 | 2 | 5 | 0.4 | 0.001935 | BP | [induction of positive chemotaxis](http://www.godatabase.org/cgi-bin/amigo/go.cgi?view=details&search_constraint=terms&depth=0&query=GO:0050930) |
| GO:0030225 | 2 | 5 | 0.4 | 0.001935 | BP | [macrophage differentiation](http://www.godatabase.org/cgi-bin/amigo/go.cgi?view=details&search_constraint=terms&depth=0&query=GO:0030225) |
| GO:0006878 | 2 | 5 | 0.4 | 0.001935 | BP | [copper ion homeostasis](http://www.godatabase.org/cgi-bin/amigo/go.cgi?view=details&search_constraint=terms&depth=0&query=GO:0006878) |
| GO:0030574 | 7 | 18 | 0.388889 | 2.73E-09 | BP | [collagen catabolism](http://www.godatabase.org/cgi-bin/amigo/go.cgi?view=details&search_constraint=terms&depth=0&query=GO:0030574) |
| GO:0000270 | 7 | 21 | 0.333333 | 9.62E-09 | BP | [peptidoglycan metabolism](http://www.godatabase.org/cgi-bin/amigo/go.cgi?view=details&search_constraint=terms&depth=0&query=GO:0000270) |
| GO:0050921 | 2 | 6 | 0.333333 | 0.002875 | BP | [positive regulation of chemotaxis](http://www.godatabase.org/cgi-bin/amigo/go.cgi?view=details&search_constraint=terms&depth=0&query=GO:0050921) |
| GO:0008637 | 2 | 7 | 0.285714 | 0.003988 | BP | [apoptotic mitochondrial changes](http://www.godatabase.org/cgi-bin/amigo/go.cgi?view=details&search_constraint=terms&depth=0&query=GO:0008637) |
| GO:0050920 | 2 | 7 | 0.285714 | 0.003988 | BP | [regulation of chemotaxis](http://www.godatabase.org/cgi-bin/amigo/go.cgi?view=details&search_constraint=terms&depth=0&query=GO:0050920) |
| GO:0030201 | 2 | 7 | 0.285714 | 0.003988 | BP | [heparan sulfate proteoglycan metabolism](http://www.godatabase.org/cgi-bin/amigo/go.cgi?view=details&search_constraint=terms&depth=0&query=GO:0030201) |
| GO:0030162 | 2 | 8 | 0.25 | 0.005268 | BP | [regulation of proteolysis](http://www.godatabase.org/cgi-bin/amigo/go.cgi?view=details&search_constraint=terms&depth=0&query=GO:0030162) |
| GO:0046883 | 2 | 8 | 0.25 | 0.005268 | BP | [regulation of hormone secretion](http://www.godatabase.org/cgi-bin/amigo/go.cgi?view=details&search_constraint=terms&depth=0&query=GO:0046883) |
| GO:0006817 | 13 | 60 | 0.216667 | 1.50E-12 | BP | [phosphate transport](http://www.godatabase.org/cgi-bin/amigo/go.cgi?view=details&search_constraint=terms&depth=0&query=GO:0006817) |
| GO:0046916 | 3 | 21 | 0.142857 | 0.003062 | BP | [transition metal ion homeostasis](http://www.godatabase.org/cgi-bin/amigo/go.cgi?view=details&search_constraint=terms&depth=0&query=GO:0046916) |
| GO:0043062 | 4 | 31 | 0.129032 | 0.000899 | BP | [extracellular structure organization and biogenesis](http://www.godatabase.org/cgi-bin/amigo/go.cgi?view=details&search_constraint=terms&depth=0&query=GO:0043062) |
| GO:0030198 | 4 | 31 | 0.129032 | 0.000899 | BP | [extracellular matrix organization and biogenesis](http://www.godatabase.org/cgi-bin/amigo/go.cgi?view=details&search_constraint=terms&depth=0&query=GO:0030198) |
| GO:0015698 | 13 | 111 | 0.117117 | 4.74E-09 | BP | [inorganic anion transport](http://www.godatabase.org/cgi-bin/amigo/go.cgi?view=details&search_constraint=terms&depth=0&query=GO:0015698) |
| GO:0006820 | 14 | 135 | 0.103704 | 5.87E-09 | BP | [anion transport](http://www.godatabase.org/cgi-bin/amigo/go.cgi?view=details&search_constraint=terms&depth=0&query=GO:0006820) |
| GO:0016066 | 3 | 29 | 0.103448 | 0.007748 | BP | [cellular defense response (sensu Vertebrata)](http://www.godatabase.org/cgi-bin/amigo/go.cgi?view=details&search_constraint=terms&depth=0&query=GO:0016066) |
| GO:0040012 | 3 | 31 | 0.096774 | 0.009337 | BP | [regulation of locomotion](http://www.godatabase.org/cgi-bin/amigo/go.cgi?view=details&search_constraint=terms&depth=0&query=GO:0040012) |
| GO:0051270 | 3 | 31 | 0.096774 | 0.009337 | BP | [regulation of cell motility](http://www.godatabase.org/cgi-bin/amigo/go.cgi?view=details&search_constraint=terms&depth=0&query=GO:0051270) |
| GO:0043122 | 7 | 74 | 0.094595 | 7.97E-05 | BP | [regulation of I-kappaB kinase/NF-kappaB cascade](http://www.godatabase.org/cgi-bin/amigo/go.cgi?view=details&search_constraint=terms&depth=0&query=GO:0043122) |
| GO:0043123 | 6 | 68 | 0.088235 | 0.000384 | BP | [positive regulation of I-kappaB kinase/NF-kappaB cascade](http://www.godatabase.org/cgi-bin/amigo/go.cgi?view=details&search_constraint=terms&depth=0&query=GO:0043123) |
| GO:0008544 | 6 | 74 | 0.081081 | 0.000606 | BP | [epidermis development](http://www.godatabase.org/cgi-bin/amigo/go.cgi?view=details&search_constraint=terms&depth=0&query=GO:0008544) |
| GO:0007249 | 8 | 101 | 0.079208 | 8.77E-05 | BP | [I-kappaB kinase/NF-kappaB cascade](http://www.godatabase.org/cgi-bin/amigo/go.cgi?view=details&search_constraint=terms&depth=0&query=GO:0007249) |
| GO:0007398 | 6 | 83 | 0.072289 | 0.001114 | BP | [ectoderm development](http://www.godatabase.org/cgi-bin/amigo/go.cgi?view=details&search_constraint=terms&depth=0&query=GO:0007398) |
| GO:0009888 | 12 | 174 | 0.068966 | 6.16E-06 | BP | [tissue development](http://www.godatabase.org/cgi-bin/amigo/go.cgi?view=details&search_constraint=terms&depth=0&query=GO:0009888) |
| GO:0009967 | 6 | 87 | 0.068966 | 0.001423 | BP | [positive regulation of signal transduction](http://www.godatabase.org/cgi-bin/amigo/go.cgi?view=details&search_constraint=terms&depth=0&query=GO:0009967) |
| GO:0050954 | 6 | 90 | 0.066667 | 0.001695 | BP | [sensory perception of mechanical stimulus](http://www.godatabase.org/cgi-bin/amigo/go.cgi?view=details&search_constraint=terms&depth=0&query=GO:0050954) |
| GO:0007605 | 6 | 90 | 0.066667 | 0.001695 | BP | [sensory perception of sound](http://www.godatabase.org/cgi-bin/amigo/go.cgi?view=details&search_constraint=terms&depth=0&query=GO:0007605) |
| GO:0001501 | 9 | 136 | 0.066176 | 0.000128 | BP | [skeletal development](http://www.godatabase.org/cgi-bin/amigo/go.cgi?view=details&search_constraint=terms&depth=0&query=GO:0001501) |
| GO:0016477 | 6 | 93 | 0.064516 | 0.002005 | BP | [cell migration](http://www.godatabase.org/cgi-bin/amigo/go.cgi?view=details&search_constraint=terms&depth=0&query=GO:0016477) |
| GO:0007155 | 29 | 520 | 0.055769 | 1.27E-10 | BP | [cell adhesion](http://www.godatabase.org/cgi-bin/amigo/go.cgi?view=details&search_constraint=terms&depth=0&query=GO:0007155) |
| GO:0006935 | 6 | 111 | 0.054054 | 0.004845 | BP | [chemotaxis](http://www.godatabase.org/cgi-bin/amigo/go.cgi?view=details&search_constraint=terms&depth=0&query=GO:0006935) |
| GO:0042330 | 6 | 111 | 0.054054 | 0.004845 | BP | [taxis](http://www.godatabase.org/cgi-bin/amigo/go.cgi?view=details&search_constraint=terms&depth=0&query=GO:0042330) |
| GO:0007626 | 6 | 116 | 0.051724 | 0.005997 | BP | [locomotory behavior](http://www.godatabase.org/cgi-bin/amigo/go.cgi?view=details&search_constraint=terms&depth=0&query=GO:0007626) |
| GO:0006928 | 11 | 218 | 0.050459 | 0.000259 | BP | [cell motility](http://www.godatabase.org/cgi-bin/amigo/go.cgi?view=details&search_constraint=terms&depth=0&query=GO:0006928) |
| GO:0040011 | 11 | 218 | 0.050459 | 0.000259 | BP | [locomotion](http://www.godatabase.org/cgi-bin/amigo/go.cgi?view=details&search_constraint=terms&depth=0&query=GO:0040011) |
| GO:0051674 | 11 | 218 | 0.050459 | 0.000259 | BP | [localization of cell](http://www.godatabase.org/cgi-bin/amigo/go.cgi?view=details&search_constraint=terms&depth=0&query=GO:0051674) |
| GO:0051603 | 7 | 139 | 0.05036 | 0.003538 | BP | [proteolysis during cellular protein catabolism](http://www.godatabase.org/cgi-bin/amigo/go.cgi?view=details&search_constraint=terms&depth=0&query=GO:0051603) |
| GO:0044257 | 7 | 140 | 0.05 | 0.003681 | BP | [cellular protein catabolism](http://www.godatabase.org/cgi-bin/amigo/go.cgi?view=details&search_constraint=terms&depth=0&query=GO:0044257) |
| GO:0030163 | 7 | 161 | 0.043478 | 0.00783 | BP | [protein catabolism](http://www.godatabase.org/cgi-bin/amigo/go.cgi?view=details&search_constraint=terms&depth=0&query=GO:0030163) |
| GO:0007243 | 10 | 251 | 0.039841 | 0.002947 | BP | [protein kinase cascade](http://www.godatabase.org/cgi-bin/amigo/go.cgi?view=details&search_constraint=terms&depth=0&query=GO:0007243) |
| GO:0044262 | 10 | 270 | 0.037037 | 0.004941 | BP | [cellular carbohydrate metabolism](http://www.godatabase.org/cgi-bin/amigo/go.cgi?view=details&search_constraint=terms&depth=0&query=GO:0044262) |
| GO:0048513 | 18 | 510 | 0.035294 | 0.000295 | BP | [organ development](http://www.godatabase.org/cgi-bin/amigo/go.cgi?view=details&search_constraint=terms&depth=0&query=GO:0048513) |
| GO:0006508 | 16 | 469 | 0.034115 | 0.000939 | BP | [proteolysis](http://www.godatabase.org/cgi-bin/amigo/go.cgi?view=details&search_constraint=terms&depth=0&query=GO:0006508) |
| GO:0009611 | 12 | 356 | 0.033708 | 0.004551 | BP | [response to wounding](http://www.godatabase.org/cgi-bin/amigo/go.cgi?view=details&search_constraint=terms&depth=0&query=GO:0009611) |
| GO:0009613 | 16 | 493 | 0.032454 | 0.001583 | BP | [response to pest, pathogen or parasite](http://www.godatabase.org/cgi-bin/amigo/go.cgi?view=details&search_constraint=terms&depth=0&query=GO:0009613) |
| GO:0051707 | 16 | 498 | 0.032129 | 0.001756 | BP | [response to other organism](http://www.godatabase.org/cgi-bin/amigo/go.cgi?view=details&search_constraint=terms&depth=0&query=GO:0051707) |
| GO:0006955 | 22 | 718 | 0.030641 | 0.000444 | BP | [immune response](http://www.godatabase.org/cgi-bin/amigo/go.cgi?view=details&search_constraint=terms&depth=0&query=GO:0006955) |
| GO:0006811 | 16 | 542 | 0.02952 | 0.004083 | BP | [ion transport](http://www.godatabase.org/cgi-bin/amigo/go.cgi?view=details&search_constraint=terms&depth=0&query=GO:0006811) |
| GO:0009607 | 24 | 829 | 0.028951 | 0.000544 | BP | [response to biotic stimulus](http://www.godatabase.org/cgi-bin/amigo/go.cgi?view=details&search_constraint=terms&depth=0&query=GO:0009607) |
| GO:0048522 | 15 | 521 | 0.028791 | 0.006773 | BP | [positive regulation of cellular process](http://www.godatabase.org/cgi-bin/amigo/go.cgi?view=details&search_constraint=terms&depth=0&query=GO:0048522) |
| GO:0048519 | 20 | 716 | 0.027933 | 0.002529 | BP | [negative regulation of biological process](http://www.godatabase.org/cgi-bin/amigo/go.cgi?view=details&search_constraint=terms&depth=0&query=GO:0048519) |
| GO:0006952 | 22 | 797 | 0.027604 | 0.001766 | BP | [defense response](http://www.godatabase.org/cgi-bin/amigo/go.cgi?view=details&search_constraint=terms&depth=0&query=GO:0006952) |
| GO:0043118 | 17 | 625 | 0.0272 | 0.006976 | BP | [negative regulation of physiological process](http://www.godatabase.org/cgi-bin/amigo/go.cgi?view=details&search_constraint=terms&depth=0&query=GO:0043118) |
| GO:0007275 | 43 | 1585 | 0.027129 | 8.81E-06 | BP | [development](http://www.godatabase.org/cgi-bin/amigo/go.cgi?view=details&search_constraint=terms&depth=0&query=GO:0007275) |
| GO:0048523 | 18 | 666 | 0.027027 | 0.005886 | BP | [negative regulation of cellular process](http://www.godatabase.org/cgi-bin/amigo/go.cgi?view=details&search_constraint=terms&depth=0&query=GO:0048523) |
| GO:0050874 | 39 | 1635 | 0.023853 | 0.000461 | BP | [organismal physiological process](http://www.godatabase.org/cgi-bin/amigo/go.cgi?view=details&search_constraint=terms&depth=0&query=GO:0050874) |
| GO:0050896 | 37 | 1737 | 0.021301 | 0.005271 | BP | [response to stimulus](http://www.godatabase.org/cgi-bin/amigo/go.cgi?view=details&search_constraint=terms&depth=0&query=GO:0050896) |
| GO:0017106 | 2 | 3 | 0.666667 | 0.000518 | MF | [activin inhibitor activity](http://www.godatabase.org/cgi-bin/amigo/go.cgi?view=details&search_constraint=terms&depth=0&query=GO:0017106) |
| GO:0008133 | 3 | 5 | 0.6 | 2.23E-05 | MF | [collagenase activity](http://www.godatabase.org/cgi-bin/amigo/go.cgi?view=details&search_constraint=terms&depth=0&query=GO:0008133) |
| GO:0008147 | 2 | 4 | 0.5 | 0.001028 | MF | [structural constituent of bone](http://www.godatabase.org/cgi-bin/amigo/go.cgi?view=details&search_constraint=terms&depth=0&query=GO:0008147) |
| GO:0005518 | 4 | 15 | 0.266667 | 3.59E-05 | MF | [collagen binding](http://www.godatabase.org/cgi-bin/amigo/go.cgi?view=details&search_constraint=terms&depth=0&query=GO:0005518) |
| GO:0005201 | 13 | 78 | 0.166667 | 2.29E-11 | MF | [extracellular matrix structural constituent](http://www.godatabase.org/cgi-bin/amigo/go.cgi?view=details&search_constraint=terms&depth=0&query=GO:0005201) |
| GO:0004222 | 10 | 84 | 0.119048 | 1.43E-07 | MF | [metalloendopeptidase activity](http://www.godatabase.org/cgi-bin/amigo/go.cgi?view=details&search_constraint=terms&depth=0&query=GO:0004222) |
| GO:0008201 | 7 | 63 | 0.111111 | 1.83E-05 | MF | [heparin binding](http://www.godatabase.org/cgi-bin/amigo/go.cgi?view=details&search_constraint=terms&depth=0&query=GO:0008201) |
| GO:0005539 | 8 | 80 | 0.1 | 1.00E-05 | MF | [glycosaminoglycan binding](http://www.godatabase.org/cgi-bin/amigo/go.cgi?view=details&search_constraint=terms&depth=0&query=GO:0005539) |
| GO:0030247 | 8 | 82 | 0.097561 | 1.21E-05 | MF | [polysaccharide binding](http://www.godatabase.org/cgi-bin/amigo/go.cgi?view=details&search_constraint=terms&depth=0&query=GO:0030247) |
| GO:0001871 | 8 | 90 | 0.088889 | 2.40E-05 | MF | [pattern binding](http://www.godatabase.org/cgi-bin/amigo/go.cgi?view=details&search_constraint=terms&depth=0&query=GO:0001871) |
| GO:0005507 | 4 | 50 | 0.08 | 0.004238 | MF | [copper ion binding](http://www.godatabase.org/cgi-bin/amigo/go.cgi?view=details&search_constraint=terms&depth=0&query=GO:0005507) |
| GO:0008237 | 10 | 139 | 0.071942 | 1.49E-05 | MF | [metallopeptidase activity](http://www.godatabase.org/cgi-bin/amigo/go.cgi?view=details&search_constraint=terms&depth=0&query=GO:0008237) |
| GO:0005125 | 9 | 190 | 0.047368 | 0.000936 | MF | [cytokine activity](http://www.godatabase.org/cgi-bin/amigo/go.cgi?view=details&search_constraint=terms&depth=0&query=GO:0005125) |
| GO:0030246 | 9 | 193 | 0.046632 | 0.001045 | MF | [carbohydrate binding](http://www.godatabase.org/cgi-bin/amigo/go.cgi?view=details&search_constraint=terms&depth=0&query=GO:0030246) |
| GO:0005198 | 22 | 542 | 0.04059 | 2.30E-06 | MF | [structural molecule activity](http://www.godatabase.org/cgi-bin/amigo/go.cgi?view=details&search_constraint=terms&depth=0&query=GO:0005198) |
| GO:0004175 | 12 | 305 | 0.039344 | 0.000719 | MF | [endopeptidase activity](http://www.godatabase.org/cgi-bin/amigo/go.cgi?view=details&search_constraint=terms&depth=0&query=GO:0004175) |
| GO:0008233 | 15 | 418 | 0.035885 | 0.000413 | MF | [peptidase activity](http://www.godatabase.org/cgi-bin/amigo/go.cgi?view=details&search_constraint=terms&depth=0&query=GO:0008233) |
| GO:0005509 | 22 | 659 | 0.033384 | 4.99E-05 | MF | [calcium ion binding](http://www.godatabase.org/cgi-bin/amigo/go.cgi?view=details&search_constraint=terms&depth=0&query=GO:0005509) |
| GO:0005102 | 18 | 555 | 0.032432 | 0.000369 | MF | [receptor binding](http://www.godatabase.org/cgi-bin/amigo/go.cgi?view=details&search_constraint=terms&depth=0&query=GO:0005102) |
| GO:0004871 | 38 | 1932 | 0.019669 | 0.005781 | MF | [signal transducer activity](http://www.godatabase.org/cgi-bin/amigo/go.cgi?view=details&search_constraint=terms&depth=0&query=GO:0004871) |
| GO:0005515 | 70 | 3944 | 0.017748 | 0.001198 | MF | [protein binding](http://www.godatabase.org/cgi-bin/amigo/go.cgi?view=details&search_constraint=terms&depth=0&query=GO:0005515) |
| GO:0005578 | 34 | 250 | 0.136 | 1.73E-24 | CC | [proteinaceous extracellular matrix](http://www.godatabase.org/cgi-bin/amigo/go.cgi?view=details&search_constraint=terms&depth=0&query=GO:0005578) |
| GO:0031012 | 34 | 254 | 0.133858 | 2.96E-24 | CC | [extracellular matrix](http://www.godatabase.org/cgi-bin/amigo/go.cgi?view=details&search_constraint=terms&depth=0&query=GO:0031012) |
| GO:0044421 | 48 | 644 | 0.074534 | 5.66E-23 | CC | [extracellular region part](http://www.godatabase.org/cgi-bin/amigo/go.cgi?view=details&search_constraint=terms&depth=0&query=GO:0044421) |
| GO:0005576 | 56 | 945 | 0.059259 | 3.43E-22 | CC | [extracellular region](http://www.godatabase.org/cgi-bin/amigo/go.cgi?view=details&search_constraint=terms&depth=0&query=GO:0005576) |
| GO:0044420 | 18 | 83 | 0.216867 | 5.29E-17 | CC | [extracellular matrix part](http://www.godatabase.org/cgi-bin/amigo/go.cgi?view=details&search_constraint=terms&depth=0&query=GO:0044420) |
| GO:0005581 | 13 | 32 | 0.40625 | 1.33E-16 | CC | [collagen](http://www.godatabase.org/cgi-bin/amigo/go.cgi?view=details&search_constraint=terms&depth=0&query=GO:0005581) |
| GO:0005583 | 7 | 11 | 0.636364 | 2.94E-11 | CC | [fibrillar collagen](http://www.godatabase.org/cgi-bin/amigo/go.cgi?view=details&search_constraint=terms&depth=0&query=GO:0005583) |
| GO:0005604 | 9 | 53 | 0.169811 | 4.35E-08 | CC | [basement membrane](http://www.godatabase.org/cgi-bin/amigo/go.cgi?view=details&search_constraint=terms&depth=0&query=GO:0005604) |
| GO:0005615 | 22 | 436 | 0.050459 | 1.64E-07 | CC | [extracellular space](http://www.godatabase.org/cgi-bin/amigo/go.cgi?view=details&search_constraint=terms&depth=0&query=GO:0005615) |
| GO:0043256 | 4 | 9 | 0.444444 | 4.46E-06 | CC | [laminin complex](http://www.godatabase.org/cgi-bin/amigo/go.cgi?view=details&search_constraint=terms&depth=0&query=GO:0043256) |
| GO:0005587 | 3 | 5 | 0.6 | 2.66E-05 | CC | [collagen type IV](http://www.godatabase.org/cgi-bin/amigo/go.cgi?view=details&search_constraint=terms&depth=0&query=GO:0005587) |
| GO:0030935 | 3 | 6 | 0.5 | 5.27E-05 | CC | [sheet-forming collagen](http://www.godatabase.org/cgi-bin/amigo/go.cgi?view=details&search_constraint=terms&depth=0&query=GO:0030935) |
| GO:0005610 | 2 | 2 | 1 | 0.00019615 | CC | [laminin-5 complex](http://www.godatabase.org/cgi-bin/amigo/go.cgi?view=details&search_constraint=terms&depth=0&query=GO:0005610) |
| GO:0005605 | 4 | 27 | 0.148148 | 0.00051054 | CC | [basal lamina](http://www.godatabase.org/cgi-bin/amigo/go.cgi?view=details&search_constraint=terms&depth=0&query=GO:0005605) |
| GO:0005584 | 2 | 3 | 0.666667 | 0.00058303 | CC | [collagen type I](http://www.godatabase.org/cgi-bin/amigo/go.cgi?view=details&search_constraint=terms&depth=0&query=GO:0005584) |
| GO:0005588 | 2 | 3 | 0.666667 | 0.00058303 | CC | [collagen type V](http://www.godatabase.org/cgi-bin/amigo/go.cgi?view=details&search_constraint=terms&depth=0&query=GO:0005588) |
| GO:0005606 | 2 | 7 | 0.285714 | 0.00393282 | CC | [laminin-1 complex](http://www.godatabase.org/cgi-bin/amigo/go.cgi?view=details&search_constraint=terms&depth=0&query=GO:0005606) |
| GO:0019898 | 4 | 60 | 0.066667 | 0.00992652 | CC | [extrinsic to membrane](http://www.godatabase.org/cgi-bin/amigo/go.cgi?view=details&search_constraint=terms&depth=0&query=GO:0019898) |
| GO:0005578 | 34 | 250 | 0.136 | 1.73E-24 | CC | [proteinaceous extracellular matrix](http://www.godatabase.org/cgi-bin/amigo/go.cgi?view=details&search_constraint=terms&depth=0&query=GO:0005578) |

* p value < 0.01.
