# Supplementary material for: Transcriptomic dissection of tongue squamous cell carcinoma
Source: BMC Genomics. 2008 Feb 6;9:69. doi: 10.1186/1471-2164-9-69 (PMC2262071; doi:10.1186/1471-2164-9-69)
Supplement: Additional file 4 — Supplement Table S4: Suppressed Biological Processes (BP), Molecular Functions (MF) and Cellular Components (CC) in OTSCC. The table showing the complete list of the suppressed biological processes (BP), molecular functions (MF) and cellular components (CC) in OTSCC (p value < 0.01). [file 1471-2164-9-69-S4.doc]

**Supplement Table S4: Suppressed Biological Processes (BP), Molecular Functions (MF) and Cellular Components (CC) in OTSCC ***

| **GO ID** | **Sig Genes** | **Genes in GO** | **Proportion of Change** | **P value** | **GO category** | **GO Term** |
| --- | --- | --- | --- | --- | --- | --- |
| GO:0043163 | 2 | 2 | 1 | 0.000157 | BP | [cell envelope organization and biogenesis](http://www.godatabase.org/cgi-bin/amigo/go.cgi?view=details&search_constraint=terms&depth=0&query=GO:0043163) |
| GO:0001942 | 2 | 3 | 0.666667 | 0.000468 | BP | [hair follicle development](http://www.godatabase.org/cgi-bin/amigo/go.cgi?view=details&search_constraint=terms&depth=0&query=GO:0001942) |
| GO:0045229 | 2 | 3 | 0.666667 | 0.000468 | BP | [external encapsulating structure organization and biogenesis](http://www.godatabase.org/cgi-bin/amigo/go.cgi?view=details&search_constraint=terms&depth=0&query=GO:0045229) |
| GO:0035315 | 2 | 3 | 0.666667 | 0.000468 | BP | [hair cell differentiation](http://www.godatabase.org/cgi-bin/amigo/go.cgi?view=details&search_constraint=terms&depth=0&query=GO:0035315) |
| GO:0042303 | 2 | 4 | 0.5 | 0.000929 | BP | [molting cycle](http://www.godatabase.org/cgi-bin/amigo/go.cgi?view=details&search_constraint=terms&depth=0&query=GO:0042303) |
| GO:0006067 | 2 | 4 | 0.5 | 0.000929 | BP | [ethanol metabolism](http://www.godatabase.org/cgi-bin/amigo/go.cgi?view=details&search_constraint=terms&depth=0&query=GO:0006067) |
| GO:0006069 | 2 | 4 | 0.5 | 0.000929 | BP | [ethanol oxidation](http://www.godatabase.org/cgi-bin/amigo/go.cgi?view=details&search_constraint=terms&depth=0&query=GO:0006069) |
| GO:0042633 | 2 | 4 | 0.5 | 0.000929 | BP | [hair cycle](http://www.godatabase.org/cgi-bin/amigo/go.cgi?view=details&search_constraint=terms&depth=0&query=GO:0042633) |
| GO:0019369 | 2 | 5 | 0.4 | 0.001535 | BP | [arachidonic acid metabolism](http://www.godatabase.org/cgi-bin/amigo/go.cgi?view=details&search_constraint=terms&depth=0&query=GO:0019369) |
| GO:0042554 | 2 | 5 | 0.4 | 0.001535 | BP | [superoxide release](http://www.godatabase.org/cgi-bin/amigo/go.cgi?view=details&search_constraint=terms&depth=0&query=GO:0042554) |
| GO:0009913 | 5 | 14 | 0.357143 | 5.33E-07 | BP | [epidermal cell differentiation](http://www.godatabase.org/cgi-bin/amigo/go.cgi?view=details&search_constraint=terms&depth=0&query=GO:0009913) |
| GO:0048730 | 5 | 15 | 0.333333 | 7.92E-07 | BP | [epidermis morphogenesis](http://www.godatabase.org/cgi-bin/amigo/go.cgi?view=details&search_constraint=terms&depth=0&query=GO:0048730) |
| GO:0031424 | 4 | 12 | 0.333333 | 1.10E-05 | BP | [keratinization](http://www.godatabase.org/cgi-bin/amigo/go.cgi?view=details&search_constraint=terms&depth=0&query=GO:0031424) |
| GO:0030855 | 3 | 10 | 0.3 | 0.000219 | BP | [epithelial cell differentiation](http://www.godatabase.org/cgi-bin/amigo/go.cgi?view=details&search_constraint=terms&depth=0&query=GO:0030855) |
| GO:0019370 | 3 | 12 | 0.25 | 0.000395 | BP | [leukotriene biosynthesis](http://www.godatabase.org/cgi-bin/amigo/go.cgi?view=details&search_constraint=terms&depth=0&query=GO:0019370) |
| GO:0042743 | 2 | 8 | 0.25 | 0.004192 | BP | [hydrogen peroxide metabolism](http://www.godatabase.org/cgi-bin/amigo/go.cgi?view=details&search_constraint=terms&depth=0&query=GO:0042743) |
| GO:0042744 | 2 | 8 | 0.25 | 0.004192 | BP | [hydrogen peroxide catabolism](http://www.godatabase.org/cgi-bin/amigo/go.cgi?view=details&search_constraint=terms&depth=0&query=GO:0042744) |
| GO:0048729 | 5 | 21 | 0.238095 | 5.05E-06 | BP | [tissue morphogenesis](http://www.godatabase.org/cgi-bin/amigo/go.cgi?view=details&search_constraint=terms&depth=0&query=GO:0048729) |
| GO:0002009 | 3 | 13 | 0.230769 | 0.000508 | BP | [morphogenesis of an epithelium](http://www.godatabase.org/cgi-bin/amigo/go.cgi?view=details&search_constraint=terms&depth=0&query=GO:0002009) |
| GO:0042542 | 2 | 9 | 0.222222 | 0.005346 | BP | [response to hydrogen peroxide](http://www.godatabase.org/cgi-bin/amigo/go.cgi?view=details&search_constraint=terms&depth=0&query=GO:0042542) |
| GO:0006691 | 3 | 14 | 0.214286 | 0.000641 | BP | [leukotriene metabolism](http://www.godatabase.org/cgi-bin/amigo/go.cgi?view=details&search_constraint=terms&depth=0&query=GO:0006691) |
| GO:0030216 | 4 | 19 | 0.210526 | 8.02E-05 | BP | [keratinocyte differentiation](http://www.godatabase.org/cgi-bin/amigo/go.cgi?view=details&search_constraint=terms&depth=0&query=GO:0030216) |
| GO:0043450 | 3 | 15 | 0.2 | 0.000794 | BP | [alkene biosynthesis](http://www.godatabase.org/cgi-bin/amigo/go.cgi?view=details&search_constraint=terms&depth=0&query=GO:0043450) |
| GO:0051262 | 2 | 11 | 0.181818 | 0.008034 | BP | [protein tetramerization](http://www.godatabase.org/cgi-bin/amigo/go.cgi?view=details&search_constraint=terms&depth=0&query=GO:0051262) |
| GO:0043449 | 3 | 17 | 0.176471 | 0.001165 | BP | [alkene metabolism](http://www.godatabase.org/cgi-bin/amigo/go.cgi?view=details&search_constraint=terms&depth=0&query=GO:0043449) |
| GO:0008544 | 10 | 74 | 0.135135 | 2.52E-08 | BP | [epidermis development](http://www.godatabase.org/cgi-bin/amigo/go.cgi?view=details&search_constraint=terms&depth=0&query=GO:0008544) |
| GO:0046456 | 3 | 23 | 0.130435 | 0.002872 | BP | [icosanoid biosynthesis](http://www.godatabase.org/cgi-bin/amigo/go.cgi?view=details&search_constraint=terms&depth=0&query=GO:0046456) |
| GO:0006805 | 3 | 24 | 0.125 | 0.003252 | BP | [xenobiotic metabolism](http://www.godatabase.org/cgi-bin/amigo/go.cgi?view=details&search_constraint=terms&depth=0&query=GO:0006805) |
| GO:0007398 | 10 | 83 | 0.120482 | 7.74E-08 | BP | [ectoderm development](http://www.godatabase.org/cgi-bin/amigo/go.cgi?view=details&search_constraint=terms&depth=0&query=GO:0007398) |
| GO:0051259 | 3 | 25 | 0.12 | 0.003662 | BP | [protein oligomerization](http://www.godatabase.org/cgi-bin/amigo/go.cgi?view=details&search_constraint=terms&depth=0&query=GO:0051259) |
| GO:0009410 | 3 | 27 | 0.111111 | 0.004572 | BP | [response to xenobiotic stimulus](http://www.godatabase.org/cgi-bin/amigo/go.cgi?view=details&search_constraint=terms&depth=0&query=GO:0009410) |
| GO:0006690 | 3 | 33 | 0.090909 | 0.008074 | BP | [icosanoid metabolism](http://www.godatabase.org/cgi-bin/amigo/go.cgi?view=details&search_constraint=terms&depth=0&query=GO:0006690) |
| GO:0006633 | 4 | 48 | 0.083333 | 0.003038 | BP | [fatty acid biosynthesis](http://www.godatabase.org/cgi-bin/amigo/go.cgi?view=details&search_constraint=terms&depth=0&query=GO:0006633) |
| GO:0016053 | 4 | 55 | 0.072727 | 0.004978 | BP | [organic acid biosynthesis](http://www.godatabase.org/cgi-bin/amigo/go.cgi?view=details&search_constraint=terms&depth=0&query=GO:0016053) |
| GO:0046394 | 4 | 55 | 0.072727 | 0.004978 | BP | [carboxylic acid biosynthesis](http://www.godatabase.org/cgi-bin/amigo/go.cgi?view=details&search_constraint=terms&depth=0&query=GO:0046394) |
| GO:0009888 | 10 | 174 | 0.057471 | 6.56E-05 | BP | [tissue development](http://www.godatabase.org/cgi-bin/amigo/go.cgi?view=details&search_constraint=terms&depth=0&query=GO:0009888) |
| GO:0006959 | 7 | 149 | 0.04698 | 0.002713 | BP | [humoral immune response](http://www.godatabase.org/cgi-bin/amigo/go.cgi?view=details&search_constraint=terms&depth=0&query=GO:0006959) |
| GO:0042592 | 7 | 169 | 0.04142 | 0.005426 | BP | [homeostasis](http://www.godatabase.org/cgi-bin/amigo/go.cgi?view=details&search_constraint=terms&depth=0&query=GO:0042592) |
| GO:0006118 | 11 | 269 | 0.040892 | 0.000565 | BP | [electron transport](http://www.godatabase.org/cgi-bin/amigo/go.cgi?view=details&search_constraint=terms&depth=0&query=GO:0006118) |
| GO:0006091 | 15 | 468 | 0.032051 | 0.000763 | BP | [generation of precursor metabolites and energy](http://www.godatabase.org/cgi-bin/amigo/go.cgi?view=details&search_constraint=terms&depth=0&query=GO:0006091) |
| GO:0030154 | 14 | 471 | 0.029724 | 0.00234 | BP | [cell differentiation](http://www.godatabase.org/cgi-bin/amigo/go.cgi?view=details&search_constraint=terms&depth=0&query=GO:0030154) |
| GO:0007275 | 34 | 1585 | 0.021451 | 0.000875 | BP | [development](http://www.godatabase.org/cgi-bin/amigo/go.cgi?view=details&search_constraint=terms&depth=0&query=GO:0007275) |
| GO:0004024 | 2 | 4 | 0.5 | 0.000895 | MF | [alcohol dehydrogenase activity, zinc-dependent](http://www.godatabase.org/cgi-bin/amigo/go.cgi?view=details&search_constraint=terms&depth=0&query=GO:0004024) |
| GO:0003810 | 2 | 6 | 0.333333 | 0.002201 | MF | [protein-glutamine gamma-glutamyltransferase activity](http://www.godatabase.org/cgi-bin/amigo/go.cgi?view=details&search_constraint=terms&depth=0&query=GO:0003810) |
| GO:0004030 | 2 | 6 | 0.333333 | 0.002201 | MF | [aldehyde dehydrogenase [NAD(P)+] activity](http://www.godatabase.org/cgi-bin/amigo/go.cgi?view=details&search_constraint=terms&depth=0&query=GO:0004030) |
| GO:0030280 | 2 | 7 | 0.285714 | 0.003057 | MF | [structural constituent of epidermis](http://www.godatabase.org/cgi-bin/amigo/go.cgi?view=details&search_constraint=terms&depth=0&query=GO:0030280) |
| GO:0004022 | 2 | 8 | 0.25 | 0.004043 | MF | [alcohol dehydrogenase activity](http://www.godatabase.org/cgi-bin/amigo/go.cgi?view=details&search_constraint=terms&depth=0&query=GO:0004022) |
| GO:0004029 | 2 | 8 | 0.25 | 0.004043 | MF | [aldehyde dehydrogenase (NAD) activity](http://www.godatabase.org/cgi-bin/amigo/go.cgi?view=details&search_constraint=terms&depth=0&query=GO:0004029) |
| GO:0050381 | 4 | 22 | 0.181818 | 0.000137 | MF | [unspecific monooxygenase activity](http://www.godatabase.org/cgi-bin/amigo/go.cgi?view=details&search_constraint=terms&depth=0&query=GO:0050381) |
| GO:0005149 | 2 | 11 | 0.181818 | 0.007751 | MF | [interleukin-1 receptor binding](http://www.godatabase.org/cgi-bin/amigo/go.cgi?view=details&search_constraint=terms&depth=0&query=GO:0005149) |
| GO:0016755 | 2 | 11 | 0.181818 | 0.007751 | MF | [transferase activity, transferring amino-acyl groups](http://www.godatabase.org/cgi-bin/amigo/go.cgi?view=details&search_constraint=terms&depth=0&query=GO:0016755) |
| GO:0015020 | 3 | 18 | 0.166667 | 0.001314 | MF | [glucuronosyltransferase activity](http://www.godatabase.org/cgi-bin/amigo/go.cgi?view=details&search_constraint=terms&depth=0&query=GO:0015020) |
| GO:0016712 | 4 | 27 | 0.148148 | 0.000313 | MF | [oxidoreductase activity, acting on paired donors, with incorporation or reduction of molecular oxygen, reduced flavin or flavoprotein as one donor, and incorporation of one atom of oxygen](http://www.godatabase.org/cgi-bin/amigo/go.cgi?view=details&search_constraint=terms&depth=0&query=GO:0016712) |
| GO:0019825 | 4 | 34 | 0.117647 | 0.000775 | MF | [oxygen binding](http://www.godatabase.org/cgi-bin/amigo/go.cgi?view=details&search_constraint=terms&depth=0&query=GO:0019825) |
| GO:0004497 | 6 | 74 | 0.081081 | 0.000295 | MF | [monooxygenase activity](http://www.godatabase.org/cgi-bin/amigo/go.cgi?view=details&search_constraint=terms&depth=0&query=GO:0004497) |
| GO:0016705 | 5 | 93 | 0.053763 | 0.005822 | MF | [oxidoreductase activity, acting on paired donors, with incorporation or reduction of molecular oxygen](http://www.godatabase.org/cgi-bin/amigo/go.cgi?view=details&search_constraint=terms&depth=0&query=GO:0016705) |
| GO:0004252 | 6 | 114 | 0.052632 | 0.002845 | MF | [serine-type endopeptidase activity](http://www.godatabase.org/cgi-bin/amigo/go.cgi?view=details&search_constraint=terms&depth=0&query=GO:0004252) |
| GO:0008236 | 6 | 124 | 0.048387 | 0.004312 | MF | [serine-type peptidase activity](http://www.godatabase.org/cgi-bin/amigo/go.cgi?view=details&search_constraint=terms&depth=0&query=GO:0008236) |
| GO:0016491 | 16 | 542 | 0.02952 | 0.001014 | MF | [oxidoreductase activity](http://www.godatabase.org/cgi-bin/amigo/go.cgi?view=details&search_constraint=terms&depth=0&query=GO:0016491) |
| GO:0005198 | 15 | 542 | 0.027675 | 0.002749 | MF | [structural molecule activity](http://www.godatabase.org/cgi-bin/amigo/go.cgi?view=details&search_constraint=terms&depth=0&query=GO:0005198) |
| GO:0005792 | 10 | 127 | 0.07874 | 4.83E-06 | CC | [microsome](http://www.godatabase.org/cgi-bin/amigo/go.cgi?view=details&search_constraint=terms&depth=0&query=GO:0005792) |
| GO:0042598 | 10 | 127 | 0.07874 | 4.83E-06 | CC | [vesicular fraction](http://www.godatabase.org/cgi-bin/amigo/go.cgi?view=details&search_constraint=terms&depth=0&query=GO:0042598) |
| GO:0005576 | 28 | 945 | 0.02963 | 1.79E-05 | CC | [extracellular region](http://www.godatabase.org/cgi-bin/amigo/go.cgi?view=details&search_constraint=terms&depth=0&query=GO:0005576) |
| GO:0005882 | 7 | 76 | 0.092105 | 4.97E-05 | CC | [intermediate filament](http://www.godatabase.org/cgi-bin/amigo/go.cgi?view=details&search_constraint=terms&depth=0&query=GO:0005882) |
| GO:0045111 | 7 | 76 | 0.092105 | 4.97E-05 | CC | [intermediate filament cytoskeleton](http://www.godatabase.org/cgi-bin/amigo/go.cgi?view=details&search_constraint=terms&depth=0&query=GO:0045111) |
| GO:0005783 | 20 | 605 | 0.033058 | 7.82E-05 | CC | [endoplasmic reticulum](http://www.godatabase.org/cgi-bin/amigo/go.cgi?view=details&search_constraint=terms&depth=0&query=GO:0005783) |
| GO:0044421 | 20 | 644 | 0.031056 | 0.00018275 | CC | [extracellular region part](http://www.godatabase.org/cgi-bin/amigo/go.cgi?view=details&search_constraint=terms&depth=0&query=GO:0044421) |
| GO:0001533 | 3 | 15 | 0.2 | 0.0008287 | CC | [cornified envelope](http://www.godatabase.org/cgi-bin/amigo/go.cgi?view=details&search_constraint=terms&depth=0&query=GO:0001533) |
| GO:0005615 | 13 | 436 | 0.029817 | 0.00375165 | CC | [extracellular space](http://www.godatabase.org/cgi-bin/amigo/go.cgi?view=details&search_constraint=terms&depth=0&query=GO:0005615) |
| GO:0016324 | 3 | 26 | 0.115385 | 0.00427416 | CC | [apical plasma membrane](http://www.godatabase.org/cgi-bin/amigo/go.cgi?view=details&search_constraint=terms&depth=0&query=GO:0016324) |
| GO:0005856 | 18 | 758 | 0.023747 | 0.00764758 | CC | [cytoskeleton](http://www.godatabase.org/cgi-bin/amigo/go.cgi?view=details&search_constraint=terms&depth=0&query=GO:0005856) |
| GO:0005913 | 2 | 11 | 0.181818 | 0.00826182 | CC | [cell-cell adherens junction](http://www.godatabase.org/cgi-bin/amigo/go.cgi?view=details&search_constraint=terms&depth=0&query=GO:0005913) |
| GO:0005624 | 15 | 591 | 0.025381 | 0.00829604 | CC | [membrane fraction](http://www.godatabase.org/cgi-bin/amigo/go.cgi?view=details&search_constraint=terms&depth=0&query=GO:0005624) |

* p value < 0.01.
