# Supplementary material for: Transcriptomic dissection of tongue squamous cell carcinoma
Source: BMC Genomics. 2008 Feb 6;9:69. doi: 10.1186/1471-2164-9-69 (PMC2262071; doi:10.1186/1471-2164-9-69)
Supplement: Additional file 5 — Supplement Table S5: Expression values of genes that constitute the altered biological processes (listed in Table 2) in OTSCC. The table showing the statistics on expression values of genes that constitute the altered biological processes in OTSCC. [file 1471-2164-9-69-S5.doc]

**Supplement Table S5: Expression values of genes that constitute the altered biological processes (identified in Table 2) in OTSCC.**

| **GO term** | **Entrz ID** | **Public ID** | **Gene name** | **Affymetrix array probesets** | **P value** | **Fold change** |
| --- | --- | --- | --- | --- | --- | --- |
| **GO:0006817** |  |  |  |  |  |  |
|  | 712 | NM_015991 | complement component 1, q subcomponent, A chain | 218232_at | 0.694008 | 1.117225 |
|  | 713 | NM_000491 | complement component 1, q subcomponent, B chain | 202953_at | 0.25178 | 1.411419 |
|  | 1277 | K01228 | collagen, type I, alpha 1 | 202310_s_at  202311_s_at  202312_s_at  217430_x_at | 0.000451 | 3.169439 |
|  | 1278 | AA788711 | collagen, type I, alpha 2 | 202403_s_at  202404_s_at | 9.15E-07 | 4.683783 |
|  | 1280 | X06268 | collagen, type II, alpha 1 | 213492_at  217404_s_at | 0.137717 | 1.094428 |
|  | 1281 | AI813758 | collagen, type III, alpha 1 | 201852_x_at  211161_s_at  215076_s_at  215077_at | 0.000391 | 2.98564 |
|  | 1282 | AI922605 | collagen, type IV, alpha 1 | 211980_at  211981_at | 2.62E-07 | 3.893552 |
|  | 1284 | X05610 | collagen, type IV, alpha 2 | 211964_at  211966_at | 0.000214 | 2.503115 |
|  | 1285 | M81379 | collagen, type IV, alpha 3 | 214641_at  216367_at  216368_s_at  216893_s_at  216896_at  216898_s_at  222073_at | 0.334793 | 0.957114 |
|  | 1286 | D17391 | collagen, type IV, alpha 4 | 214602_at | 0.864741 | 0.988459 |
|  | 1287 | AW052179 | collagen, type IV, alpha 5 | 213110_s_at | 0.00011 | 2.415379 |
|  | 1288 | BC005305 | collagen, type IV, alpha 6 | 210945_at  211473_s_at  213992_at | 1.99E-06 | 3.757882 |
|  | 1289 | AI130969 | collagen, type V, alpha 1 | 203325_s_at  212488_at  212489_at | 0.000308 | 3.06461 |
|  | 1290 | AL575735 | collagen, type V, alpha 2 | 221729_at  221730_at | 5.60E-06 | 3.907361 |
|  | 1291 | AI141603 | collagen, type VI, alpha 1 | 212091_s_at  212937_s_at  212938_at  212939_at  212940_at  213428_s_at  214200_s_at  216904_at | 0.011346 | 1.810167 |
|  | 1292 | AY029208 | collagen, type VI, alpha 2 | 209156_s_at  213290_at | 0.05277 | 1.633671 |
|  | 1293 | NM_004369 | collagen, type VI, alpha 3 | 201438_at | 0.000214 | 2.704057 |
|  | 1294 | NM_000094 | collagen, type VII, alpha 1 | 204136_at  217312_s_at | 0.010739 | 1.671633 |
|  | 1295 | BE877796 | collagen, type VIII, alpha 1 | 214587_at  221152_at | 0.278551 | 1.062679 |
|  | 1296 | AI806793 | collagen, type VIII, alpha 2 | 221900_at  52651_at | 0.137283 | 1.340419 |
|  | 1297 | NM_001851 | collagen, type IX, alpha 1 | 222008_at | 0.210803 | 0.934907 |
|  | 1298 | AI733465 | collagen, type IX, alpha 2 | 213622_at | 0.209472 | 1.140138 |
|  | 1299 | NM_001853 | collagen, type IX, alpha 3 | 204724_s_at | 0.334761 | 1.10454 |
|  | 1300 | AI376003 | collagen, type X, alpha 1 | 205941_s_at  217428_s_at | 0.008159 | 2.15613 |
|  | 1301 | NM_001854 | collagen, type XI, alpha 1 | 204320_at  37892_at | 0.000934 | 4.006326 |
|  | 1302 | AL031228 | collagen, type XI, alpha 2 | 213870_at  216993_s_at | 0.742656 | 0.977381 |
|  | 1305 | NM_005203 | collagen, type XIII, alpha 1 | 208535_x_at  211343_s_at  211809_x_at | 0.000127 | 1.678778 |
|  | 1306 | NM_001855 | collagen, type XV, alpha 1 | 203477_at | 0.993565 | 0.996869 |
|  | 1307 | NM_001856 | collagen, type XVI, alpha 1 | 204345_at | 0.003771 | 1.789617 |
|  | 1308 | NM_000494 | collagen, type XVII, alpha 1 | 204636_at | 0.344833 | 1.303442 |
|  | 1310 | D38163 | collagen, type XIX, alpha 1 | 211011_at | 0.668478 | 0.979636 |
|  | 1896 | NM_001399 | ectodysplasin A | 206217_at  211127_x_at  211128_at  211129_x_at  211130_x_at  211131_s_at | 0.071133 | 0.85509 |
|  | 2219 | NM_002003 | ficolin (collagen/fibrinogen domain containing) 1 | 205237_at | 0.081189 | 0.855187 |
|  | 2220 | NM_004108 | ficolin (collagen/fibrinogen domain containing lectin) 2 (hucolin) | 207804_s_at  208439_s_at | 0.880896 | 0.98909 |
|  | 4153 | NM_000242 | mannose-binding lectin (protein C) 2, soluble (opsonic defect) | 207256_at | 0.669502 | 0.983205 |
|  | 4481 | NM_002445 | macrophage scavenger receptor 1 | 208422_at  208423_s_at  211887_x_at  214770_at | 0.056692 | 1.118654 |
|  | 6436 | NM_006926 | surfactant, pulmonary-associated protein A2 | 218835_at | 0.101306 | 0.896712 |
|  | 6441 | NM_003019 | surfactant, pulmonary-associated protein D | 214199_at | 0.793962 | 1.025784 |
|  | 6568 | NM_005074 | solute carrier family 17 (sodium phosphate), member 1 | 206872_at | 0.847914 | 0.98425 |
|  | 6569 | NM_003052 | solute carrier family 34 (sodium phosphate), member 1 | 208177_at  217530_at | 0.362109 | 0.949961 |
|  | 6574 | NM_005415 | solute carrier family 20 (phosphate transporter), member 1 | 201920_at | 8.13E-05 | 2.001347 |
|  | 6575 | NM_006749 | solute carrier family 20 (phosphate transporter), member 2 | 202744_at | 0.804408 | 1.033331 |
|  | 7373 | BF449063 | collagen, type XIV, alpha 1 (undulin) | 212865_s_at  216865_at  216866_s_at | 0.005555 | 0.485316 |
|  | 8292 | AF057036 | collagen-like tail subunit (single strand of homotrimer) of asymmetric acetylcholinesterase | 206073_at | 0.398967 | 0.956039 |
|  | 8685 | NM_006770 | macrophage receptor with collagenous structure | 205819_at | 0.044219 | 0.826603 |
|  | 9370 | NM_004797 | adiponectin, C1Q and collagen domain containing | 207175_at | 0.00035 | 0.322687 |
|  | 10568 | AF146796 | solute carrier family 34 (sodium phosphate), member 2 | 204124_at | 0.713645 | 0.959985 |
|  | 10882 | NM_006688 | complement component 1, q subcomponent-like 1 | 205575_at  214346_at | 0.38423 | 0.970402 |
|  | 11117 | NM_007046 | elastin microfibril interfacer 1 | 204163_at | 0.943815 | 1.01713 |
|  | 50509 | NM_015719 | collagen, type V, alpha 3 | 218975_at  52255_s_at | 0.010483 | 1.62808 |
|  | 51435 | NM_016240 | scavenger receptor class A, member 3 | 219416_at | 0.0996 | 0.87037 |
|  | 55339 | NM_018383 | WD repeat domain 33 | 218851_s_at | 0.775587 | 0.972147 |
|  | 56172 | NM_019847 | ankylosis, progressive homolog (mouse) | 220076_at | 0.657154 | 0.964869 |
|  | 78989 | NM_024027 | collectin sub-family member 11 | 219873_at | 0.742731 | 1.054527 |
|  | 80781 | NM_030582 | collagen, type XVIII, alpha 1 | 209081_s_at  209082_s_at | 0.331873 | 1.175597 |
|  | 81578 | NM_030820 | collagen, type XXI, alpha 1 | 208096_s_at | 0.277988 | 0.807739 |
|  | 114897 | NM_030968 | C1q and tumor necrosis factor related protein 1 | 220975_s_at | 0.982412 | 1.003153 |
|  | 114899 | NM_030945 | C1q and tumor necrosis factor related protein 3 | 220988_s_at | 0.618142 | 0.889209 |
|  | 129080 | AL031186 | EMI domain containing 1 | 213779_at | 0.10014 | 0.877686 |
|  | 136227 | BC003528 | EMI domain containing 2 | 210920_x_at | 0.806096 | 1.015063 |
| **GO:0030574** |  |  |  |  |  |  |
|  | 4312 | NM_002421 | matrix metallopeptidase 1 | 204475_at | 0 | 57.61528 |
|  | 4313 | NM_004530 | matrix metallopeptidase 2 | 201069_at | 0.109232 | 1.443179 |
|  | 4314 | NM_002422 | matrix metallopeptidase 3 | 205828_at | 1.01E-08 | 8.434561 |
|  | 4316 | NM_002423 | matrix metallopeptidase 7 | 204259_at | 0.004907 | 2.827885 |
|  | 4317 | NM_002424 | matrix metallopeptidase 8 | 207329_at | 0.796281 | 1.013264 |
|  | 4318 | NM_004994 | matrix metallopeptidase 9 | 203936_s_at | 5.85E-05 | 4.079094 |
|  | 4319 | NM_002425 | matrix metallopeptidase 10 | 205680_at | 9.95E-06 | 8.448239 |
|  | 4320 | AI761713 | matrix metallopeptidase 11 | 203876_s_at  203877_at  203878_s_at  213602_s_at | 0.000847 | 2.027893 |
|  | 4322 | NM_002427 | matrix metallopeptidase 13 | 205959_at | 0.000712 | 3.791227 |
|  | 4325 | U79292 | matrix metallopeptidase 16 | 207012_at  207013_s_at  208166_at  208167_s_at | 0.111695 | 0.931528 |
|  | 4327 | NM_002429 | matrix metallopeptidase 19 | 204574_s_at  204575_s_at | 0.440357 | 1.084575 |
|  | 5184 | NM_000285 | peptidase D | 202108_at | 0.460222 | 0.924964 |
|  | 5645 | NM_002770 | protease, serine, 2 (trypsin 2) | 205402_x_at | 0.031454 | 0.761082 |
|  | 5653 | NM_002774 | kallikrein 6 (neurosin, zyme) | 204733_at | 0.42179 | 0.734167 |
|  | 5657 | NM_002777 | proteinase 3 | 207341_at | 0.265608 | 0.955804 |
|  | 9508 | AB002364 | ADAM metallopeptidase with thrombospondin type 1 motif, 3 | 214913_at | 0.585811 | 1.050908 |
|  | 9509 | NM_014244 | ADAM metallopeptidase with thrombospondin type 1 motif, 2 | 214454_at  214535_s_at | 0.245065 | 1.190667 |
|  | 56547 | NM_021801 | matrix metallopeptidase 26 | 220541_at | 0.239356 | 0.940011 |
| **GO:0043123** |  |  |  |  |  |  |
|  | 329 | NM_001166 | baculoviral IAP repeat-containing 2 | 202076_at | 0.050641 | 1.437152 |
|  | 356 | D38122 | Fas ligand (TNF superfamily, member 6) | 210865_at  211333_s_at | 0.724342 | 0.970241 |
|  | 387 | BC001360 | ras homolog gene family, member A | 200059_s_at | 0.386313 | 0.898884 |
|  | 684 | NM_004335 | bone marrow stromal cell antigen 2 | 201641_at | 3.85E-06 | 3.425675 |
|  | 834 | AI719655 | caspase 1, apoptosis-related cysteine peptidase | 206011_at  209970_x_at  211366_x_at  211367_s_at  211368_s_at | 0.116807 | 1.221104 |
|  | 841 | NM_001228 | caspase 8, apoptosis-related cysteine peptidase | 207686_s_at  213373_s_at | 0.577467 | 1.071557 |
|  | 958 | NM_001250 | CD40 molecule, TNF receptor superfamily member 5 | 205153_s_at  215346_at  222292_at  35150_at | 0.165258 | 1.123585 |
|  | 1893 | U65932 | extracellular matrix protein 1 | 209365_s_at | 8.24E-06 | 0.30796 |
|  | 1894 | NM_018098 | epithelial cell transforming sequence 2 oncogene | 219787_s_at | 3.01E-05 | 2.562727 |
|  | 1902 | AW269335 | endothelial differentiation, lysophosphatidic acid G-protein-coupled receptor, 2 | 204036_at  204037_at  204038_s_at | 0.040218 | 0.691976 |
|  | 1936 | NM_001960 | eukaryotic translation elongation factor 1 delta (guanine nucleotide exchange protein) | 203113_s_at  213087_s_at  214394_x_at  214395_x_at | 0.429789 | 1.138522 |
|  | 2149 | NM_001992 | coagulation factor II (thrombin) receptor | 203989_x_at | 0.002385 | 1.40368 |
|  | 2280 | NM_000801 | FK506 binding protein 1A, 12kDa | 200709_at  210186_s_at  210187_at  214119_s_at | 0.264094 | 1.154305 |
|  | 2316 | NM_001456 | filamin A, alpha | 200859_x_at  213746_s_at  214752_x_at | 0.075061 | 1.279765 |
|  | 2697 | NM_000165 | gap junction protein, alpha 1, 43kDa | 201667_at | 0.000642 | 2.201027 |
|  | 3162 | NM_002133 | heme oxygenase (decycling) 1 | 203665_at | 0.668878 | 0.909028 |
|  | 3357 | NM_000867 | 5-hydroxytryptamine (serotonin) receptor 2B | 206638_at | 0.952291 | 0.993284 |
|  | 3956 | NM_002305 | lectin, galactoside-binding, soluble, 1 (galectin 1) | 201105_at  216405_at | 0.010631 | 1.933721 |
|  | 3965 | NM_009587 | lectin, galactoside-binding, soluble, 9 (galectin 9) | 203236_s_at | 0.551562 | 1.072954 |
|  | 4055 | NM_002342 | lymphotoxin beta receptor | 203005_at | 0.51607 | 1.070563 |
|  | 4215 | BF971923 | mitogen-activated protein kinase kinase kinase 3 | 203514_at | 0.089224 | 0.868835 |
|  | 4615 | U70451 | myeloid differentiation primary response gene (88) | 209124_at | 0.406262 | 0.898756 |
|  | 5494 | NM_021003 | protein phosphatase 1A (formerly 2C), magnesium-dependent, alpha isoform | 203966_s_at  210407_at | 0.848609 | 0.97918 |
|  | 5536 | NM_006247 | protein phosphatase 5, catalytic subunit | 201979_s_at  215705_at | 0.401301 | 0.947503 |
|  | 5966 | NM_002908 | v-rel reticuloendotheliosis viral oncogene homolog (avian) | 206035_at  206036_s_at | 0.010357 | 1.236384 |
|  | 5970 | NM_021975 | v-rel reticuloendotheliosis viral oncogene homolog A, nuclear factor of kappa light polypeptide gene enhancer in B-cells 3, p65 (avian) | 201783_s_at  209878_s_at | 0.673264 | 1.043217 |
|  | 6398 | BF939675 | secreted and transmembrane 1 | 213716_s_at | 0.651924 | 1.097055 |
|  | 6574 | NM_005415 | solute carrier family 20 (phosphate transporter), member 1 | 201920_at | 8.13E-05 | 2.001347 |
|  | 7105 | AF053453 | tetraspanin 6 | 209108_at  209109_s_at | 0.019623 | 0.630055 |
|  | 7132 | NM_001065 | tumor necrosis factor receptor superfamily, member 1A | 207643_s_at | 0.195707 | 1.151119 |
|  | 7188 | NM_004619 | TNF receptor-associated factor 5 | 204352_at | 0.634581 | 1.11724 |
|  | 7189 | NM_004620 | TNF receptor-associated factor 6 | 205558_at | 0.728266 | 0.973601 |
|  | 7334 | BE262760 | ubiquitin-conjugating enzyme E2N (UBC13 homolog, yeast) | 201523_x_at  201524_x_at  212751_at | 0.671302 | 1.068778 |
|  | 7335 | BG164064 | ubiquitin-conjugating enzyme E2 variant 1 | 201001_s_at | 0.997061 | 0.999138 |
|  | 8717 | L41690 | TNFRSF1A-associated via death domain | 1729_at  205641_s_at  213443_at | 0.459948 | 0.964831 |
|  | 8737 | U50062 | receptor (TNFRSF)-interacting serine-threonine kinase 1 | 209941_at | 0.971001 | 1.004817 |
|  | 8743 | U57059 | tumor necrosis factor (ligand) superfamily, member 10 | 202687_s_at  202688_at  214329_x_at | 0.009091 | 2.075019 |
|  | 8767 | AF027706 | receptor-interacting serine-threonine kinase 2 | 209544_at  209545_s_at | 5.91E-07 | 1.531808 |
|  | 8772 | NM_003824 | Fas (TNFRSF6)-associated via death domain | 202535_at | 0.035257 | 1.74592 |
|  | 8795 | BC001281 | tumor necrosis factor receptor superfamily, member 10b | 209294_x_at  209295_at  210405_x_at | 0.020843 | 1.467668 |
|  | 8837 | NM_003879 | CASP8 and FADD-like apoptosis regulator | 208485_x_at  209508_x_at  209939_x_at  210563_x_at  210564_x_at  211316_x_at  211317_s_at  211862_x_at  214486_x_at  214618_at  217654_at | 0.213905 | 0.872372 |
|  | 8915 | AF082283 | B-cell CLL/lymphoma 10 | 205263_at | 0.891105 | 1.018986 |
|  | 9218 | AF154847 | VAMP (vesicle-associated membrane protein)-associated protein A, 33kDa | 208780_x_at | 0.443097 | 1.104272 |
|  | 9516 | AB034747 | lipopolysaccharide-induced TNF factor | 200704_at  200706_s_at | 0.000144 | 1.612123 |
|  | 9641 | NM_014002 | inhibitor of kappa light polypeptide gene enhancer in B-cells, kinase epsilon | 204549_at  214398_s_at | 0.490815 | 1.054215 |
|  | 10206 | NM_005798 | ret finger protein 2 | 203659_s_at | 0.000609 | 0.659116 |
|  | 10342 | NM_006070 | TRK-fused gene | 217839_at  221871_s_at | 0.003542 | 1.314298 |
|  | 10392 | NM_006092 | caspase recruitment domain family, member 4 | 221073_s_at | 0.321439 | 0.905233 |
|  | 10475 | AU157590 | tripartite motif-containing 38 | 203567_s_at  203568_s_at  203610_s_at | 0.147059 | 0.863075 |
|  | 10758 | AW296296 | TRAF3 interacting protein 2 | 202987_at  215411_s_at | 0.51333 | 1.079069 |
|  | 10769 | NM_006622 | polo-like kinase 2 (Drosophila) | 201939_at | 0.836871 | 1.065178 |
|  | 10892 | NM_006785 | mucosa associated lymphoid tissue lymphoma translocation gene 1 | 208309_s_at  210017_at  210018_x_at | 0.140492 | 0.831997 |
|  | 22984 | AL547263 | programmed cell death 11 | 212422_at  212424_at | 0.181607 | 1.157734 |
|  | 23118 | AF241230 | mitogen-activated protein kinase kinase kinase 7 interacting protein 2 | 210284_s_at  212184_s_at | 0.602295 | 1.062797 |
|  | 23390 | AI621223 | zinc finger, DHHC-type containing 17 | 212982_at  216871_at  217486_s_at | 0.301091 | 0.95135 |
|  | 23636 | NM_016553 | nucleoporin 62kDa | 202153_s_at  207740_s_at  214935_at | 0.005316 | 1.294489 |
|  | 27032 | AF225981 | ATPase, Ca++ transporting, type 2C, member 1 | 209934_s_at  209935_at  211137_s_at  212255_s_at | 3.51E-05 | 1.534689 |
|  | 29110 | NM_013254 | TANK-binding kinase 1 | 218520_at | 0.013983 | 1.25912 |
|  | 51026 | NM_016072 | golgi transport 1 homolog B (S. cerevisiae) | 218193_s_at | 0.000125 | 2.173545 |
|  | 51463 | NM_016334 | G protein-coupled receptor 89A | 220642_x_at  222140_s_at | 0.046558 | 1.211213 |
|  | 54503 | NM_019028 | zinc finger, DHHC-type containing 13 | 219296_at | 0.118556 | 0.733574 |
|  | 54862 | NM_017721 | coiled-coil and C2 domain containing 1A | 207083_s_at  221888_at  222137_at  58994_at | 0.187133 | 0.941674 |
|  | 56674 | NM_020644 | TMEM9 domain family, member B | 218065_s_at | 0.008331 | 0.7589 |
|  | 64127 | NM_022162 | caspase recruitment domain family, member 15 | 220066_at | 0.323042 | 1.169001 |
|  | 80762 | NM_030571 | Nedd4 family interacting protein 1 | 217800_s_at | 0.701958 | 1.071606 |
|  | 80833 | NM_014349 | apolipoprotein L, 3 | 221087_s_at | 0.758778 | 0.954484 |
|  | 81552 | NM_030796 | NA | 208091_s_at | 0.008699 | 1.566309 |
|  | 124583 | AK026161 | calcium activated nucleotidase 1 | 221732_at  46323_at | 0.361172 | 1.103801 |
| **GO:0030198** |  |  |  |  |  |  |
|  | 1284 | X05610 | collagen, type IV, alpha 2 | 211964_at  211966_at | 0.000214 | 2.503115 |
|  | 1288 | BC005305 | collagen, type IV, alpha 6 | 210945_at  211473_s_at  213992_at | 1.99E-06 | 3.757882 |
|  | 1292 | AY029208 | collagen, type VI, alpha 2 | 209156_s_at  213290_at | 0.05277 | 1.633671 |
|  | 1296 | AI806793 | collagen, type VIII, alpha 2 | 221900_at  52651_at | 0.137283 | 1.340419 |
|  | 1301 | NM_001854 | collagen, type XI, alpha 1 | 204320_at  37892_at | 0.000934 | 4.006326 |
|  | 1310 | D38163 | collagen, type XIX, alpha 1 | 211011_at | 0.668478 | 0.979636 |
|  | 1758 | NM_004407 | dentin matrix acidic phosphoprotein | 208175_s_at  217067_s_at | 0.446307 | 0.971226 |
|  | 4146 | M55683 | matrilin 1, cartilage matrix protein | 206904_at  206905_s_at | 0.280465 | 0.919798 |
|  | 4148 | NM_002381 | matrilin 3 | 206091_at | 0.025921 | 1.071193 |
|  | 9806 | AI952009 | sparc/osteonectin, cwcv and kazal-like domains proteoglycan (testican) 2 | 202523_s_at  202524_s_at | 0.40527 | 0.883446 |
|  | 11005 | NM_006846 | serine peptidase inhibitor, Kazal type 5 | 205185_at | 7.68E-06 | 0.142829 |
|  | 24144 | AL080147 | tuftelin interacting protein 11 | 202750_s_at  202751_at | 0.152073 | 1.128511 |
|  | 55790 | NM_018371 | NA | 219049_at | 0.62321 | 1.118499 |
|  | 164656 | AI912086 | transmembrane protease, serine 6 | 214955_at | 0.353066 | 0.915378 |
|  | 375790 | AI424797 | agrin | 212283_at  212285_s_at  217410_at  217419_x_at | 0.008032 | 1.430637 |
|  | 1302 | AL031228 | collagen, type XI, alpha 2 | 213870_at  216993_s_at | 0.742656 | 0.977381 |
|  | 4060 | NM_002345 | lumican | 201744_s_at | 0.000189 | 2.700033 |
|  | 7373 | BF449063 | collagen, type XIV, alpha 1 (undulin) | 212865_s_at  216865_at  216866_s_at | 0.005555 | 0.485316 |
|  | 9508 | AB002364 | ADAM metallopeptidase with thrombospondin type 1 motif, 3 | 214913_at | 0.585811 | 1.050908 |
|  | 4586 | AW192795 | mucin 5AC, oligomeric mucus/gel-forming | 214303_x_at  214385_s_at  217182_at  217187_at | 0.195465 | 0.830909 |
|  | 43 | AI190022 | acetylcholinesterase (Yt blood group) | 205377_s_at  205378_s_at  210332_at | 0.017074 | 0.872377 |
|  | 4897 | NM_005010 | neuronal cell adhesion molecule | 204105_s_at  216959_x_at | 0.716234 | 1.097302 |
|  | 5457 | NM_006237 | POU domain, class 4, transcription factor 1 | 206940_s_at  211341_at | 0.281293 | 1.052381 |
|  | 22871 | NM_014932 | neuroligin 1 | 205893_at | 0.730401 | 1.019057 |
|  | 26059 | Z38645 | ELKS/RAB6-interacting/CAST family member 2 | 213938_at | 0.101561 | 1.215406 |
|  | 56123 | NM_018933 | protocadherin beta 13 | 221450_x_at | 0.479188 | 1.059578 |
|  | 56125 | NM_018931 | protocadherin beta 11 | 208504_x_at | 0.551101 | 1.048002 |
|  | 56130 | NM_018939 | protocadherin beta 6 | 221317_x_at | 0.855172 | 0.985199 |
|  | 56132 | NM_018937 | protocadherin beta 3 | 221410_x_at | 0.682911 | 1.030152 |
|  | 1286 | D17391 | collagen, type IV, alpha 4 | 214602_at | 0.864741 | 0.988459 |
|  | 7311 | AF348700 | ubiquitin A-52 residue ribosomal protein fusion product 1 | 221700_s_at  51200_at | 0.138474 | 0.910256 |
| **GO:0050921** |  |  |  |  |  |  |
|  | 9353 | AF055585 | slit homolog 2 (Drosophila) | 209897_s_at | 0.170047 | 0.876335 |
|  | 566 | NM_001700 | azurocidin 1 (cationic antimicrobial protein 37) | 214575_s_at | 0.164347 | 0.948431 |
|  | 3576 | NM_000584 | interleukin 8 | 202859_x_at  205592_at  211506_s_at | 1.54E-06 | 5.866572 |
|  | 6696 | M83248 | secreted phosphoprotein 1 (osteopontin, bone sialoprotein I, early T-lymphocyte activation 1) | 209875_s_at | 0.002693 | 3.234513 |
|  | 7422 | AF022375 | vascular endothelial growth factor | 210512_s_at  210513_s_at  211527_x_at  212171_x_at | 0.461435 | 1.203569 |
|  | 7857 | NM_003469 | secretogranin II (chromogranin C) | 204035_at | 0.990118 | 0.996818 |
| **GO:0042554** |  |  |  |  |  |  |
|  | 239 | NM_000697 | arachidonate 12-lipoxygenase | 207206_s_at | 5.88E-05 | 0.325564 |
|  | 50506 | NM_014080 | dual oxidase 2 | 219727_at | 0.691011 | 0.902799 |
|  | 50507 | NM_016931 | NADPH oxidase 4 | 219773_at | 0.00231 | 1.679007 |
|  | 53905 | AL137592 | dual oxidase 1 | 215800_at  219597_s_at | 0.001368 | 0.428157 |
|  | 79400 | NM_024505 | NADPH oxidase, EF-hand calcium binding domain 5 | 220641_at | 0.378207 | 0.925962 |
| **GO:0031424** |  |  |  |  |  |  |
|  | 2125 | NM_001988 | envoplakin | 204503_at | 0.000555 | 0.546617 |
|  | 3713 | NM_005547 | involucrin | 214599_at | 0.347591 | 0.652665 |
|  | 4014 | NM_000427 | loricrin | 207720_at | 0.081551 | 0.534789 |
|  | 5493 | NM_002705 | periplakin | 203407_at | 0.003842 | 0.370003 |
|  | 6698 | AI923984 | small proline-rich protein 1A | 213796_at  214549_x_at | 0.281739 | 0.567933 |
|  | 6699 | NM_003125 | small proline-rich protein 1B (cornifin) | 205064_at | 0.841456 | 0.872601 |
|  | 6707 | NM_005416 | small proline-rich protein 3 | 218990_s_at | 0.002721 | 0.135765 |
|  | 7051 | NM_000359 | transglutaminase 1 (K polypeptide epidermal type I, protein-glutamine-gamma-glutamyltransferase) | 206008_at | 0.003627 | 0.322892 |
|  | 7053 | NM_003245 | transglutaminase 3 (E polypeptide, protein-glutamine-gamma-glutamyltransferase) | 206004_at | 1.61E-05 | 0.172884 |
|  | 7062 | N30878 | trichohyalin | 213780_at | 0.239835 | 0.661929 |
|  | 26239 | NM_014357 | late cornified envelope 2B | 207710_at | 0.819738 | 0.969283 |
|  | 55507 | NM_018654 | G protein-coupled receptor, family C, group 5, member D | 221297_at | 0.787031 | 0.961372 |
| **GO:0042743** |  |  |  |  |  |  |
|  | 50506 | NM_014080 | dual oxidase 2 | 219727_at | 0.691011 | 0.902799 |
|  | 53905 | AL137592 | dual oxidase 1 | 215800_at  219597_s_at | 0.001368 | 0.428157 |
|  | 847 | NM_001752 | catalase | 201432_at  211922_s_at  215573_at | 0.001062 | 0.575456 |
|  | 2878 | NM_002084 | glutathione peroxidase 3 (plasma) | 201348_at  214091_s_at | 0.000175 | 0.330296 |
|  | 4025 | U39573 | lactoperoxidase | 210682_at | 0.707146 | 0.930494 |
|  | 4353 | J02694 | myeloperoxidase | 203948_s_at  203949_at | 0.900256 | 0.98844 |
|  | 7173 | M17755 | thyroid peroxidase | 210342_s_at | 0.05499 | 0.893724 |
|  | 8288 | X14346 | eosinophil peroxidase | 214627_at | 0.804408 | 0.978845 |
| **GO:0042542** |  |  |  |  |  |  |
|  | 51022 | NM_016066 | glutaredoxin 2 | 219933_at | 0.281683 | 1.111939 |
|  | 847 | NM_001752 | catalase | 201432_at  211922_s_at  215573_at | 0.001062 | 0.575456 |
|  | 2878 | NM_002084 | glutathione peroxidase 3 (plasma) | 201348_at  214091_s_at | 0.000175 | 0.330296 |
|  | 4025 | U39573 | lactoperoxidase | 210682_at | 0.707146 | 0.930494 |
|  | 4353 | J02694 | myeloperoxidase | 203948_s_at  203949_at | 0.900256 | 0.98844 |
|  | 7173 | M17755 | thyroid peroxidase | 210342_s_at | 0.05499 | 0.893724 |
|  | 8288 | X14346 | eosinophil peroxidase | 214627_at | 0.804408 | 0.978845 |
|  | 50506 | NM_014080 | dual oxidase 2 | 219727_at | 0.691011 | 0.902799 |
|  | 53905 | AL137592 | dual oxidase 1 | 215800_at  219597_s_at | 0.001368 | 0.428157 |
| **GO:0030216** |  |  |  |  |  |  |
|  | 182 | BF056748 | jagged 1 (Alagille syndrome) | 209097_s_at  209098_s_at  209099_x_at  216268_s_at | 0.08875 | 1.344546 |
|  | 301 | NM_000700 | annexin A1 | 201012_at | 0.009232 | 0.573934 |
|  | 1475 | NM_005213 | cystatin A (stefin A) | 204971_at | 0.293623 | 0.695835 |
|  | 1832 | NM_004415 | desmoplakin | 200606_at | 0.85195 | 1.048042 |
|  | 2069 | NM_001432 | epiregulin | 205767_at | 0.942211 | 0.969797 |
|  | 2125 | NM_001988 | envoplakin | 204503_at | 0.000555 | 0.546617 |
|  | 3713 | NM_005547 | involucrin | 214599_at | 0.347591 | 0.652665 |
|  | 4014 | NM_000427 | loricrin | 207720_at | 0.081551 | 0.534789 |
|  | 5742 | NM_000962 | prostaglandin-endoperoxide synthase 1 (prostaglandin G/H synthase and cyclooxygenase) | 205127_at  205128_x_at  215813_s_at | 0.879843 | 1.053683 |
|  | 5743 | NM_000963 | prostaglandin-endoperoxide synthase 2 (prostaglandin G/H synthase and cyclooxygenase) | 204748_at | 0.030819 | 2.117054 |
|  | 6278 | NM_002963 | S100 calcium binding protein A7 (psoriasin 1) | 205916_at | 0.055073 | 3.210112 |
|  | 6698 | AI923984 | small proline-rich protein 1A | 213796_at  214549_x_at | 0.281739 | 0.567933 |
|  | 6699 | NM_003125 | small proline-rich protein 1B (cornifin) | 205064_at | 0.841456 | 0.872601 |
|  | 6702 | NM_006518 | small proline-rich protein 2C | 220664_at | 0.113162 | 0.51646 |
|  | 6707 | NM_005416 | small proline-rich protein 3 | 218990_s_at | 0.002721 | 0.135765 |
|  | 7051 | NM_000359 | transglutaminase 1 (K polypeptide epidermal type I, protein-glutamine-gamma-glutamyltransferase) | 206008_at | 0.003627 | 0.322892 |
|  | 7053 | NM_003245 | transglutaminase 3 (E polypeptide, protein-glutamine-gamma-glutamyltransferase) | 206004_at | 1.61E-05 | 0.172884 |
|  | 8796 | NM_003843 | sciellin | 206884_s_at | 4.63E-07 | 0.110991 |
|  | 7042 | BF061658 | transforming growth factor, beta 2 | 209908_s_at  209909_s_at  220406_at  220407_s_at | 0.01007 | 1.406425 |
